# Evaluation of Artificial Intelligence in Participating Structure-Based Virtual Screening for Identifying Novel Interleukin-1 Receptor Associated Kinase-1 Inhibitors

**DOI:** 10.3389/fonc.2020.01769

**Published:** 2020-09-03

**Authors:** Jinxin Che, Ruiwei Feng, Jian Gao, Hongyun Yu, Qinjie Weng, Qiaojun He, Xiaowu Dong, Jian Wu, Bo Yang

**Affiliations:** ^1^Hangzhou Institute of Innovative Medicine, College of Pharmaceutical Sciences, Zhejiang University, Hangzhou, China; ^2^College of Computer Science and Technology, Zhejiang University, Hangzhou, China; ^3^Real Doctor AI Research Centre, School of Medicine, Zhejiang University, Hangzhou, China; ^4^Innovation Institute for Artificial Intelligence in Medicine, Zhejiang University, Hangzhou, China; ^5^Cancer Center of Zhejiang University, Hangzhou, China

**Keywords:** virtual screening, artificial intelligence, machine learning, IRAK1, inhibitors

## Abstract

Interleukin-1 receptor associated kinase-1 (IRAK1) exhibits important roles in inflammation, infection, and autoimmune diseases; however, only a few inhibitors have been discovered. In this study, at first, a discriminatory structure-based virtual screening (SBVS) was employed, but only one active compound (compound **1**, IC_50_ = 2.25 μM) was identified. The low hit rate (2.63%) which derives from the weak discriminatory power of docking among high-scored molecules was observed in our virtual screening (VS) process for IRAK1 inhibitor. Furthermore, an artificial intelligence (AI) method, which employed a support vector machine (SVM) model, integrated information of molecular docking, pharmacophore scoring and molecular descriptors was constructed to enhance the traditional IRAK1-VS protocol. Using AI, it was found that VS of IRAK1 inhibitors excluded by over 50% of the inactive compounds, which could significantly improve the prediction accuracy of the SBVS model. Moreover, four active molecules (two of which exhibited comparative IC_50_ with compound **1**) were accurately identified from a set of highly similar candidates. Amongst, compounds with better activity exhibited good selectivity against IRAK4. The AI assisted workflow could serve as an effective tool for enhancement of SBVS.

## Introduction

In the process of drug discovery, hunting for lead compounds is not only a starting point, but also a very challenging task. With the emergence of comprehensive chemical databases, high throughput screening (HTS), and virtual screening (VS) have been employed for finding lead compounds from known chemicals (1). As a complementary approach to HTS (2), VS filters chemicals through ligand- or structure-based approaches by taking advantages of high-performance computers, overcomes some shortcomings of HTS, and remarkably reducing the time, money, and resources involved (3, 4). However, some problems still exist in the individual VS method. For example, the scoring functions of virtual screening are not accurate enough to predict the protein-ligand binding affinity and this leads to a high rate of false results, which needs combined strategies to improve prediction accuracy in a sequential or parallel manner (5, 6).

In recent years, artificial intelligence (AI) has offered new opportunities in drug discovery. The AI techniques that have displayed superior performances (7, 8) in finding new active chemicals include Naive Bayes, support vector machine (SVM), random forest (RF), feed-forward artificial neural networks (ANNs), and deep neural network approaches. It has been reported that the hit rate of VS can be significantly improved by combining AI methods. For example, Leong et al. developed an accurate ensemble docking scheme, which established a SVM based on combinatorial docking features and molecular descriptors to predict N-methyl-D-aspartate-receptor GluN1-ligand binding affinity (9). Tian et al. integrated ensemble molecular docking and complex-based pharmacophore searching using Naive Bayesian classification and recursive partitioning, which were of great significance in the discovery of novel ROCK inhibitors and increased the VS hit rate to 28.95% (10). Hence, the application of AI in VS seems promising.

**FIGURE 1:**
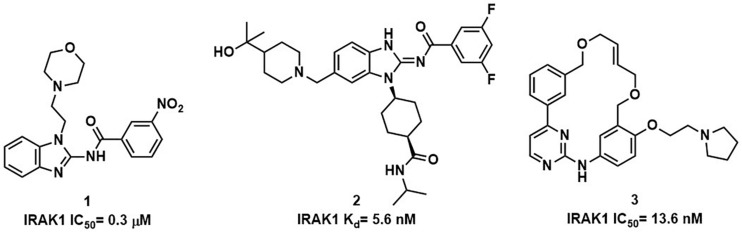
Examples of compounds with IRAK1 inhibitory activity.

Interleukin receptor associated kinase-1 (IRAK1) is a downstream member of the serine-threonine kinase interleukin receptor associated kinase (IRAK) family. Once the IL1-Rs and toll-like receptors (TLRs) are activated, IRAK4 is recruited to form the signaling complex with myeloid differentiation primary-response gene 88 (MyD88) and then IRAK1 is phosphorylated, which plays a crucial role in inflammation, infection, and autoimmune diseases (11). Researchers found that the suppression of IRAK1, either by inhibitors or RNAi, has potent activity against Waldenström’s macroglobulinemia (12), myelodysplastic syndrome (13), and certain subtypes of acute myeloid leukemia (14). However, IRAK1 selective inhibitors are rare, and most of the compounds that inhibit IRAK1 are also IRAK4 inhibitors. The benzimidazole derivative 1 shown in Figure 1 is commonly used in bio-experiment (15). In addition, researchers found that the anaplastic lymphoma kinase (ALK) inhibitor 2 (16), and Pacritinib (17), the JAK/FLT3 inhibitor 3, could be potent inhibitors of IRAK1.

As a continuation of our virtual screening work which identified new inhibitors targeting NIK, CHK1, Akt, etc. (18–24). In this study, we performed a traditional VS procedure to identify potential inhibitors of IRAK1. Using a well-designed screening process, only one compound (compound **1**, IC_50_ = 2.25 μM) was discovered, which was far from satisfactory. Considering the advantages of AI in VS, we further established a machine-learning model that combines multiple docking, complementary pharmacophore mapping, and molecular descriptors on the basis of a traditional VS workflow to increase the enrichment rate among high- scoring compounds. Training data consisted of IRAK1 inhibitors and decoys that were prepared for SVM, XGBOOST, and LGBM models. Finally, we used the SVM model that exhibited the best accuracy, to validate the activities of molecules in the post-docking stage and found that it significantly improved the performance of traditional VS and excluded over half of the false positive candidates which was predicted positive in VS but showed no activities in bioassay. Moreover, four other active compounds (compound **2**, IC_50_ = 2.32 μM; compound **3**, IC_50_ = 2.48 μM; compound **4** IC_50_ = 18.04 μM; and compound **5** IC_50_ = 23.75 μM) were identified from a series of highly similar compounds by utilizing this model, which demonstrated that the integrated VS strategy enhanced by AI was promising in the process of drug discovery.

## Materials and Methods

### Evaluation of the Docking Method

Crystal structure of human wild type IRAK1 (PDB ID: 6BFN) with its inhibitor (DL1) was downloaded from RCSB Protein Data Bank. Each chain of the protein was prepared separately including the removal of water, alternate position of residues, the addition of hydrogens, the assignment of bond orders, the optimization of H-bonds and the restrained minimization of energy using Schrödinger’s *Protein Preparation Wizard*. The receptor grids were generated using the *Receptor Grid Generation* module and all preparation parameters were set to default. The inhibitors were re-docked into their receptors to calculate the root mean square deviation (RMSD) value and compared with their original structure. Furthermore, 594 inhibitors with known IC_50_ values were collected from the ChEMBL database (Supplementary Table S1) and 150 potent inhibitors were selected after structural clustering to generate a decoy dataset using the DUD-E database (25). All 150 inhibitors and 9200 decoys were prepared using the *Ligprep* module to generate the possible ionization states and three-dimensional conformations. Lastly, the compounds were docked separately to the prepared chains of 6BFN using the SP and XP patterns in the *Ligand Docking* module. Similarly, the compounds and proteins were docked using AutoDock after preparation via *AutoDockTools* with a grid size of 54 points. The docking scores were collected to calculate the *p*-value after the docking process.

### Pharmacophore Construction

The best conformation of 6BFN_B re-docking complex was applied to construct the pharmacophore model for ligand-based VS. The pharmacophore containing at most 7 features and a receptor-based excluded volume shell was created using *Develop Pharmacophore Model* in the Schrödinger module, with the method set to E-Pharmacophore.

### Virtual Screening

A database containing 1.5 million compounds was downloaded from ChemDiv and was filtered using Lipinski’s Rule-of-Five before commencing the process of VS. Molecules which passed the filter were further aligned to the established pharmacophore model in the *Ligand Screening* module of Schrödinger so that all compounds with similar structural features could be considered for the next step. After the preparation in *Ligprep*, the molecules preserved were docked in *Ligand Docking* module under standard precision (SP) and default parameters. The top-scoring compounds were subjected to extra precision (XP) docking in order to exclude the molecules that were low-ranking in the XP mode. The compounds which underwent the entire screening protocol were clustered based on their structural similarity and selected manually for the bioactivity test.

### IRAK1 Bioactivity Test

Mobility shift assay was applied to test the bioactivity of the candidates at a concentration of 20 μM using Staurosporine as the positive control. All compounds were dissolved in 100% DMSO to yield a final concentration of 2 mM. IRAK1 kinase and kinase substrate (including ATP) were dissolved in 1 × Kinase buffer. A mixture of 250 nL of the test compound and 10 μL kinase solution were centrifuged at 1000 rpm for 30 s and incubated for 10 min at 25°C in a 384-well white plate. The plate was centrifuged at 1000 rpm and incubated for 60 min after the addition of 15 μL kinase solution (including ATP). Finally, 30 μL stop buffer was added to terminate the reaction and the conversion rate was evaluated using ELIASA (Caliper EZ Reader, Perkin Elmer) to calculate the rate of inhibition.

### SVM-Assisted Selection and Bioactivity Test

#### Machine Learning Dataset

An inhibitor dataset and a decoy dataset made up the training set of the machine- learning model. The inhibitors of IRAK1 were collected from ChEMBL Database and 150 potent inhibitors were extracted after a structural clustering. For the decoy dataset, 150 chemicals were randomly selected from the single-target compounds database of ChEMBL. Compounds in the training set were prepared and docked into each chain of 6BFN using Glide-SP, Glide-XP, and AutoDock. All docking parameters used were the same as those in the evaluation process.

A group of novel pharmacophore models were generated using the *3D QSAR Pharmacophore Generation* module of Discovery Studio Client 2.5 using the 594 inhibitors found in the ChEMBL Database. H-bond acceptors, H-bond donors, hydrophobic molecules, and aromatic compounds were considered and the upper limit of each feature was set to 5. For every molecule, at most 255 conformations were generated to find the best conformation and the maximum pharmacophores was set to 10. Since the pharmacophore models were built, the best conformations of compounds in the training dataset were generated and mapped to the pharmacophores flexibly in the *Ligand Pharmacophore Mapping* module of Discovery Studio Client 2.5. The fit value of each molecule was extracted. The compounds that failed to map onto the pharmacophore model were assigned fit values that were set to 0 uniformly.

*PaDEL v2.20* was used to calculate the 1D and 2D descriptors of the compounds in the inhibitor and decoy sets. Salts were removed and the nitro groups were standardized before the calculation.

#### Data Preprocessing

In this study, we conducted data pre-processing including index elimination and data normalization. We collected almost 1460 indices to represent each molecule, which is large for any machine learning model to analyze. Thus, we used several approaches to eliminate some indices. This process mainly consisted of the following 4 steps:

(1) Counting: We counted the value of each index. Some of the indices on the condition were excluded if more than 90% molecules had the same value which indicates that there is no crucial significance of such indices.
(2) Correlation analysis: The correlation values of all indices were calculated between any pairs. If the value was larger than 0.85, one of the pairs was excluded because of their high collinearity.
(3) *T*-test analysis: Then we calculated the *T*-test scores for the means of two indices. This test assumed that the populations had identical variances by default.
(4) Principal components analysis (PCA): Finally, we utilized PCA for dimensionality reduction.

After conducting these four steps, only 244 of the 1460 indices remained. Then, the normalization on the basis of mean and standard deviation of a batch was conducted.

#### Model Construction

In this paper, we constructed three traditional machine learning models including SVM, LGBM, and XGBoost using Scikit-learn package.

(1) Support vector machine (26) is one of the most commonly used binary classification model. Its basic principle is to defy a linear classifier in the feature space with the largest spacing. In this study, we set C as 1, using a kernel function of RBF. RBF was calculated as follows:

$$\mathrm{RBF}=\exp\,\left(-\gamma \,{\left|u-v\right|}^{2}\right)$$

Besides, we used “degree 3, coef 0” for the kernel function. We used the shrinking heuristic and probability methods. When training error was less than 1e-3, we stopped further training.
(2) LightGBM is a gradient boosting framework, based on a histogram decision tree algorithm. Using leaf-wise growth strategy with depth limitation, LightGBM model can yield better performance and prevent overfitting. In this study, we used the gbdt boosting method and set the learning rate as 0.1 in order to prevent overfitting. We constructed the classifier with 10 leaves, with “max depth 3” and “minimum child samples 31.”
(3) XGBoost uses depth-wise strategy. It first ranks all features according to their values using a pre-sorted algorithm and then splits samples. However, pre-sorted algorithm may cause additional memory space. In this study, an XGBoost model with 150 estimators, max depth 3, and minimum child weight 1 was conducted. We randomly selected 80% of samples and 80% of features to build a decision tree and used binary logistic function to learn and update parameters.

#### Metrics

In this study, to compare the performance of different models, we utilized the area under the curve (AUC) and accuracy (ACC) as metrics. AUC was defined as the area of the receiver operating characteristic (ROC) Curve, which was defined by true positive rate (TPR) and false positive rate (FPR). The TPR and FPR are given by:

$$\mathrm{TPR}=\frac{\mathrm{TP}}{\mathrm{TP}+\mathrm{FN}}$$

$$\mathrm{FPR}=\frac{\mathrm{FP}}{\mathrm{FP}+\mathrm{TN}}$$

Where TP, FP, TN, and FN were true positives, false positives, true negatives and false negatives, respectively. AUC and ACC were computed using the following equations.

$$\mathrm{AUC}=\int_{x=0}^{1}\mathrm{TPR}\,\left({\mathrm{FPR}}^{-1}\,\left(x\right)\right)\,\mathrm{dx}$$

$$\mathrm{ACC}=\frac{\mathrm{TP}+\mathrm{TN}}{\mathrm{TP}+\mathrm{TN}+\mathrm{FP}+\mathrm{FN}}$$

#### Prediction and Bioassay

Similarity search was carried out using the MolPort database. Ten chemicals, whose Tanimoto similarity was larger than 0.8 compared with compound **1**, were collected along with their multiple docking scores, pharmacophore scores, and molecular descriptors. These data were processed using the best model to predict their inhibition toward IRAK1. Candidates in the last stage of virtual screening procedure were also predicted similarly.

Lastly, compounds derived using the similarity search were bought from TargetMol and their inhibition rate was tested using the method described above. The dose-effect curve (all compounds were dissolved in DMSO and diluted 10-fold from 100 μM to 1 nM) was fitted to calculate the IC_50_ value.

### Molecular Dynamics Simulation

The binding mode of compound **1** was confirmed using molecular dynamics simulation. The parameter files of compound **1** were generated in *antechamber* module of *Amber* and was combined with 6BFN_B under ff99SB and gaff force field. After six chloride ions were added, the neutralized system was solvated in a tetrahedral box of TIP3P and the distance between box boundary and IRAK1 protein was set to 10.0 Å. At first, the energy of the entire system was relaxed using three steps: the hydrogen atoms, chloride ions, and water were optimized using 2500 steps of steepest descent minimization and 2500 steps of conjugate gradient minimization; the side chains of the protein, chloride ions, and waters were relaxed using 2500 steps of steepest descent minimization and 2500 steps of conjugate gradient minimization; the system was minimized using 2500 steps of steepest descent minimization and 2500 steps of conjugate gradient minimization. The system was heated to 300 K in 100 ps and equilibrated for 100 ps in NPT mode (temperature = 300 K; pressure = 1 atm) with the heavy atoms of the complex being restrained. Then, the whole system was equilibrated in NPT mode for 100 ps. Lastly, a five nanosecond MD simulation was carried out and the binding energy was decomposed.

## Results

### Virtual Screening

**FIGURE 2:**
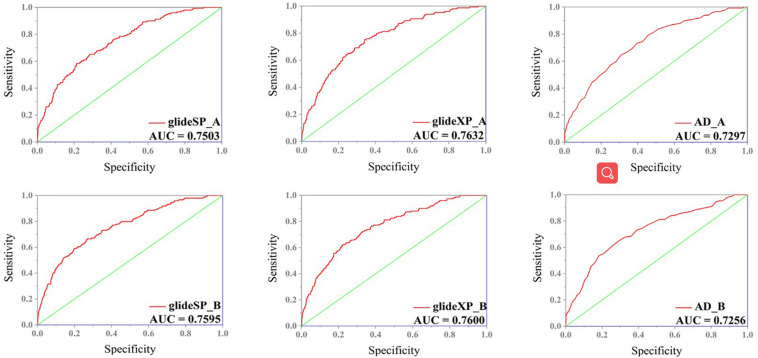
ROC curve of different combination of receptors (6BFN_A, 6BFN_B) and docking software (Glide SP, XP, and AutoDock).

**FIGURE 3:**
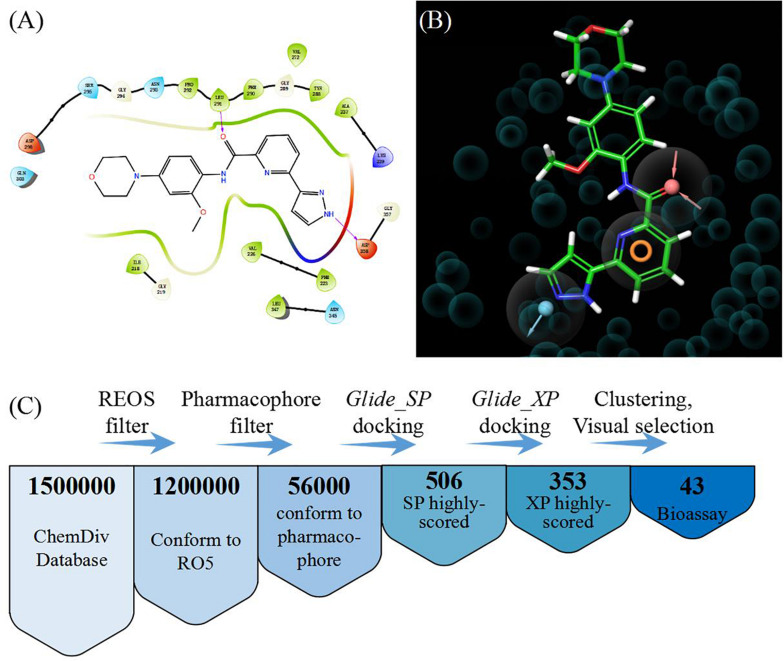
**(A)** Binding mode of the ligand (DL1) in 6BFN_B; **(B)** Pharmacophore model; and **(C)** Work flow of virtual screening.

In order to select the most effective screening method, we evaluated the performance of different proteins and molecular-docking software. For each monomer of the IRAK1 crystal structure (6BFN_A and 6BFN_B from protein data bank) (27), we docked the original ligand into the binding pocket to evaluate the reproducibility of several frequently-used docking methods. We then shortlisted 150 potent IRAK1 inhibitors and used 9200 random decoys to judge whether the docking methods and protein chains could distinguish the inhibitors from decoys effectively. As seen in Table 1, the RMSD value for each docking approach was less than 1, which indicated that all approaches could reproduce the structure of each complex accurately. The best capabilities of discrimination power were exhibited by 6BFN_B coupling with *Glide SP* and *Glide XP*, which reached the smallest *p*-value of 10^–32^ (Supplementary Figure S1). The ROC curve and the AUC are shown in Figure 2. Since the performances of the docking methods used in our study were not adequately satisfactory, we established a pharmacophore model to filter out molecules that we did not wish to pursue beyond the docking process. According to the conformation of the 6BFN_B re-docking results, we analyzed the binding mode of the IRAK1 inhibitor (Figure 3A) and constructed a pharmacophore, which contained a hydrogen-bond donor, a hydrogen-bond acceptor, and an aromatic ring, using the *Develop Pharmacophore Model* of Schrödinger which is suitable for the preliminary virtual screening and saving the time costs of calculation (Figure 3B).

**TABLE 1:** Evaluation of docking methods.

PDB_ID	Method	RMSD	Docking score	*p*_value
6BFN_A	*AutoDock*	0.5755	–10.0	8.92E−27
6BFN_B	*AutoDock*	0.5665	–9.9	4.79E−26
6BFN_A	*Glide_SP*	0.4728	–9.8	2.24E−30
6BFN_B	*Glide_SP*	0.9543	–9.3	6.87E−32
6BFN_A	*Glide_XP*	0.4742	–6.8	5.27E−32
6BFN_B	*Glide_XP*	0.9438	–6.4	4.60E−32

We collected 1.5 million chemicals from the ChemDiv database. Firstly, compounds in the database were pre-processed using a filter which could exclude chemicals that did not conform to the Lipinski’s rule of five (28). Next, we screened the remaining 1.2 million compounds in the established pharmacophore model and 56,000 molecules having a structure similar to that of the IRAK1 inhibitor were preserved. *Glide* SP docking was applied and all docked compounds were ranked by their docking scores. We selected the first 506 molecules for *Glide* XP docking in order to eliminate the compounds with lower docking scores. Thus, we obtained 353 molecules that possessed both, high docking scores and different docking precision. These compounds were clustered according to their molecular fingerprints. The most representative 43 candidates (38 compounds were purchasable) from each cluster were selected for biological assay (Figure 3C).

### Biological Evaluation

To test whether the selected molecules were active, kinase activity experiments were carried out using the mobility shift assay, in which staurosporine was chosen as the positive control. Each compound was dissolved in DMSO at a concentration of 20 μM and used in the assay (Supplementary Table S2). Compound **1** (Y041-8246) showed a moderate inhibition rate (85.5% at 20 μM) compared to staurosporine (IC_50_ = 59.29 nM; Supplementary Figure S2). The screening hit rate was 2.63% (1/38). The Tanimoto similarities between compound **1** and the known IRAK1 inhibitors were below 0.34, thus indicating that this was a new structure which bears benzofuran scaffold for IRAK1 inhibition. Moreover, structure clustering of IRAK1 inhibitors collected from ChEMBL was performed, the result indicated that benzofuran derivatives were different from any of these scaffolds (Supplementary Figure S3).

### Establishment of Machine Learning Model

**FIGURE 4:**
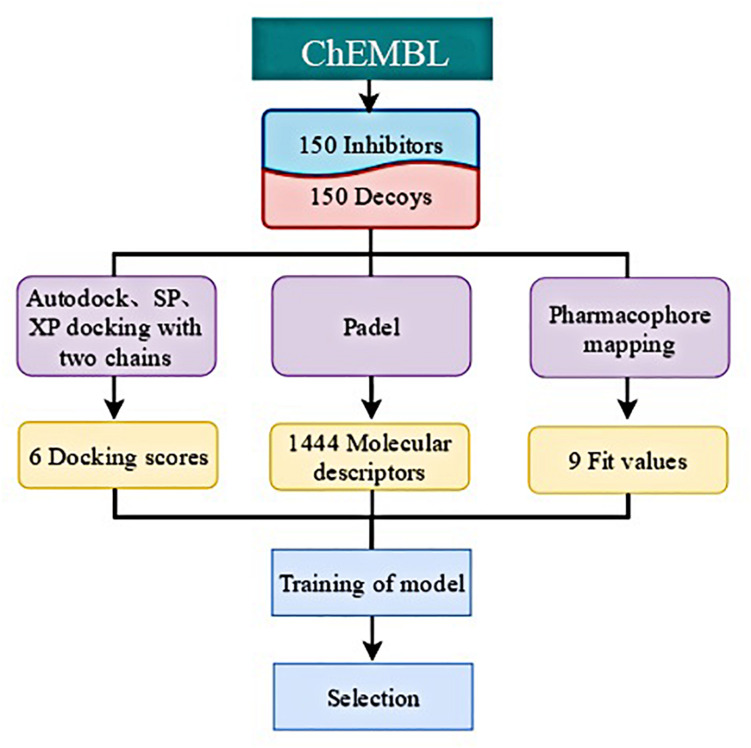
Construction of training dataset.

Considering the unsatisfactory performance of the traditional VS method with a hit rate of 2.63% in this study, which could not completely meet the demand of drug discovery, we attempted to find a better approach to discriminate lead compounds from a set of high-scored compounds. Ten molecules from the MolPort database^1^ whose Tanimoto similarities were higher than 0.8, when compared with compound **1**, were chosen to be further studied. In order to take the pharmacophore and structural information into consideration, we calculated multiple docking scores, complementary pharmacophore mapping scores, and molecular descriptors (Figure 4). The molecules in the training set consisted of 150 potent IRAK1 inhibitors and 150 decoys (Supplementary Table S3). Their docking scores were obtained using AutoDock (29), Glide SP, and Glide XP toward 6BFN_A and 6BFN_B, respectively. In this model, since it was need to construct different pharmacophore models according to known ligands, Discovery Studio was employed and all selected molecules were then mapped to pharmacophores to calculate their fit values, which demonstrated their extent of conformity to the pharmacophore models. The one- and two-dimensional molecular descriptors were calculated using *PaDEL* (30) to complement the physicochemical properties. Lastly, 6 docking scores, 9 fit values, and 1444 molecular descriptors were obtained for each compound.

Based on the characteristics of the data, we employed several traditional machine learning methods for activity prediction. In this section, we describe how data pre-processing was performed and the three machine-learning models for activity prediction was constructed. Considering the inactivity of most molecules, we used ACC and AUC to measure the performance of each model. For a fair comparison, we divided the 300 molecules into 5 groups to conduct a 5-fold cross validation. For the final test, all parameters were selected based on the best ACC performance of models on validation sets. All models were developed using python 3.7.3 with TensorFlow deep learning library and all experiments were constructed on an NVIDIA GeForce GTX 1080Ti GPU.

**FIGURE 5:**
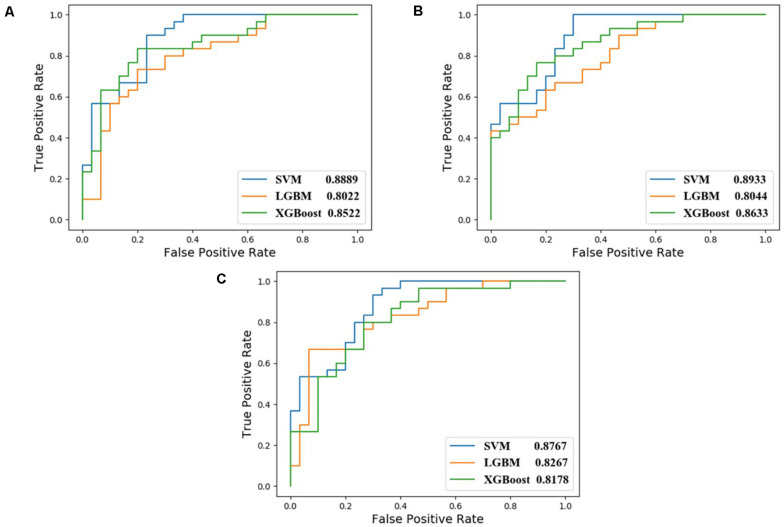
ROC curves of different combination of models and input data. **(A)** corresponds to feed model with data containing DS, PS, and MD; **(B)** for PS and MD; and **(C)** for data with MD only.

In this study, we compared the performance of each model using data with different inputs. The mean AUC and ACC scores of different models on 5-fold validation sets are shown in Table 2, different inputs (docking scores, pharmacophore mapping scores, and molecular descriptors) are presented in the first column and simply denoted as DS, PS, and MD, respectively. That is to say, the AUC and ACC results of different models in the first row are obtained by models receiving docking scores, pharmacophore mapping scores and molecular descriptors at the same time, while results in the last row are obtained by models merely receiving molecular descriptor. Obviously, a slight improvement of AUC or ACC is observed when docking and pharmacophore information were taken into consideration. Besides, it is notable in each row that SVM outperforms all the other two models. Therefore, we use SVM with all the three kind of inputs as the final model, denoted as SVM (DS+PS+MD). Table 2 and Figure 5 illustrate the performance of each combination of model and input data.

**TABLE 2:** Comparison of our three traditional machine-learning models for activity prediction.

Model	SVM		LGBM		XGBoost	
			
Data	AUC	ACC	AUC	ACC	AUC	ACC
DS+PS+MD	0.8889	0.78	0.8022	0.75	0.8522	0.77
PS+MD	0.8933	0.75	0.8044	0.72	0.8633	0.78
MD	0.8767	0.77	0.8267	0.73	0.8178	0.70

### SVM-Model Can Identify Active Compounds Accurately

**FIGURE 6:**
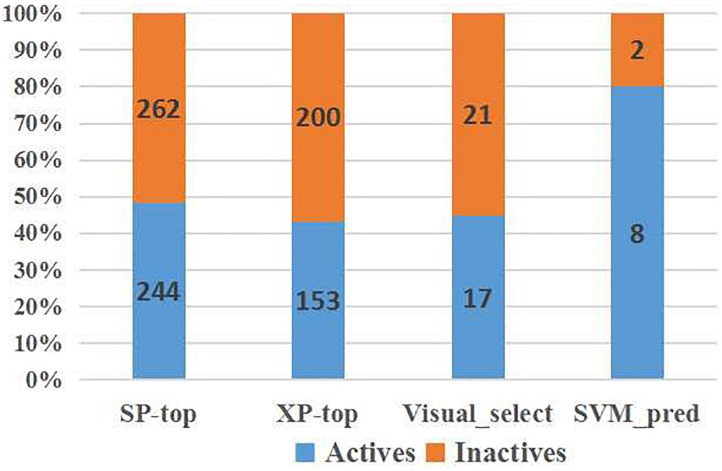
SVM model performance on IRAK1 VS candidates (top scoring compounds derived from Glide SP docking, Glide XP docking, visual selection, and SVM prediction).

To verify the practicality of the SVM (DS+PS+MD) model, we predicted the activity of molecules at later stages of VS (Figure 6). Among the molecules exhibiting superior docking scores in Glide SP and Glide XP, our model identified 51.78% selected from Glide SP, and 56.66% from Glide XP docking as inactive. Among the 38 candidates whose inhibitory had been tested, 21 inactive chemicals were accurately identified. Although there also existed several inactive candidates that were classified as active, compound **1** was picked precisely.

Ten compounds in the MolPort database with a Tanimoto similarity greater than 0.8 as compared to compound **1** were collected. Since almost all multiple docking scores appeared similar and very close (Table 3), the docking software was unable to yield accurate results. When the training information of these compounds was fed into the SVM model, the results classified two compounds as inactive, although one of them had the best average docking score. The decrease in the exclusion rate from 50 to 20% indicated that this model was competent to identify the structural features of IRAK1 inhibitors, which could thus help computational chemists select lead compounds more accurately.

**TABLE 3:** Detailed information of compounds predicted by SVM model (compound ID; docking scores from Glide SP, Glide XP, and AutoDock; average docking scores; predicted labels and IC_50_ values).

Comp_ID	SP_A	SP_B	XP_A	XP_B	AD_A	AD_B	AVG	AI_Pred	IC_50_/μ M
2	–10.25	–10.39	–11.06	–11.07	–8.1	–8.1	–9.83	1	2.32
3	–10.22	–10.28	–10.92	–10.91	–9	–9	–10.06	1	2.48
4	–9.51	–7.27	–10.58	–10.68	–11.7	–11.7	–10.24	1	18.04
5	–10.32	–10.52	–11.25	–11.88	–9.4	–9.4	–10.46	1	23.75
6	–9.95	–10.12	–11.84	–11.03	–11.2	–11.2	–10.89	0	NA^a^
7	–9.9	–9.93	–11.81	–11.01	–7.9	–7.9	–9.74	0	NA
8	–9.26	–9.66	–10.26	–10.53	–9.9	–9.9	–9.92	1	NA
9	–9.66	–9.6	–11.26	–11.57	–10	–10	–10.35	1	NA
10	–9.7	–9.33	–10.95	–10.95	–10.1	–10.1	–10.19	1	NA
11	–9.36	–6.19	–8.65	–8.89	–9.4	–9.4	–8.65	1	NA

*^*a*^NA = No Activity.*

**FIGURE 7:**
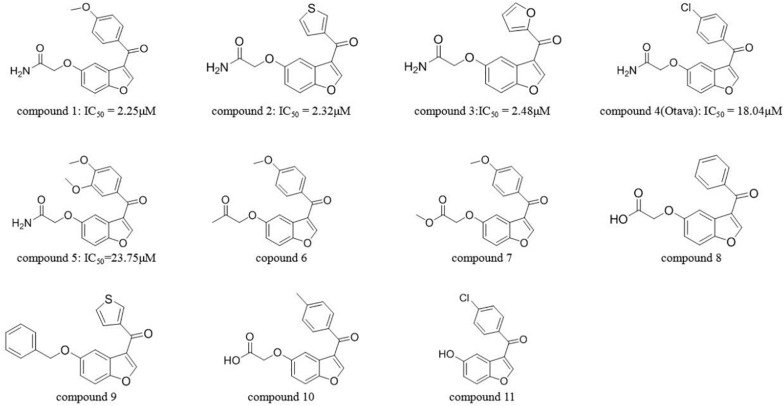
Structure of compound **1** and those predicted using SVM (compounds 2–11).

Next, the activities of these compounds were tested. Ten candidates were bought from TargetMol and their IC_50_s were determined. The initial concentration was set to 100 μM and a serial 10-fold dilution was made to 1 nM. Compounds 2 (Y041-7950) and 3 (Y041-6433) exhibited similar IRAK1 inhibition and their IC_50_ values were determined to be approximately 2 μM. Additionally, two other chemicals showed weaker inhibition and their IC_50_ values were determined to be 18.04 μM (compound **4**: 11570480 from Otava Database) and 23.75 μM, (compound **5**: Y041-7951), respectively (Figure 7 and Supplementary Figure S2). We further tested the activity against IRAK4 of the three most potent IRAK1 inhibitors such as compound **1**, **2**, and **3**. The result indicated that all the compounds showed extreme low inhibitory activities against IRAK4, which IC_50_s were > 100 μM (Supplementary Figure S4). The compounds bearing benzofuran scaffold were demonstrated an over 40-fold selectivity to IRAK4, showing its potential to be developed as selective IRAK1 inhibitor. When these compounds were used in our model, it accurately predicted the results that were determined earlier in the similarity search results. The AI model proved to be an effective tool in this prediction.

### Molecular Dynamics Simulation

**FIGURE 8:**
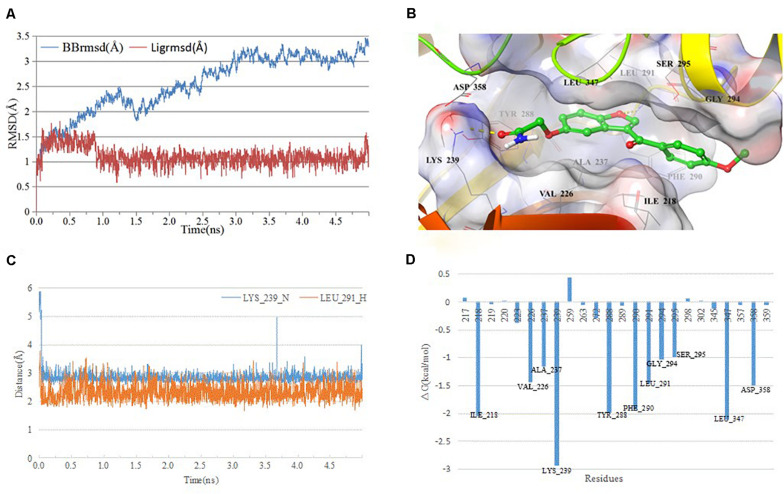
Analysis of MD simulation. **(A)** root mean square deviation (RMSD) and root mean square fluctuation (RMSF) of the IRAK1 backbone during MD simulation; **(B)** 3D-plot of the complex; **(C)** length of the hydrogen bond (compound **1**_O-Leu_291_H, compound **1**_C = O-Lys_239_N); and **(D)** plot of free energy decomposition.

Since the active compounds share same scaffold, the most potent compound **1** was chosen for the analysis of binding mode with IRAK1. In order to analyze the most stable binding pattern, we performed 5 ns molecular dynamics simulation of compound **1** using AmberTools (31). As seen in Figure 8, the RMSD value of the IRAK1 backbone and the ligand reached equilibration after 3 ns and 1 ns simulation, respectively. The average RMSF value of compound 1 was 2.47 Å, indicating a stable binding pattern. Compound 1 formed two hydrogen bonds with the H-bond donors from LYS_239 and LEU_291, and also formed stable Van der Waals interaction through hydrophobic or hydrophilic amino acid residues such as ILE_218, PHE_294, LEU_347, and ASP_358. The lengths of the H-bonds and MM-GBSA free energy confirmed that the hydrogen bond between compound **1** and LYS_239 was more stable and contributed significantly to the formation of the complex.

## Discussion and Conclusion

In this study, we discovered a novel IRAK1 inhibitor (compound **1**) through traditional virtual screening and also obtained four similar compounds which exhibited good to moderate IRAK1 activity in the AI-aided selection process. As the screening power of docking-based virtual screening was significantly weak (hit rate = 2.63%) to select hit compounds from highly scored molecules, an AI-based discriminatory virtual screening protocol was conducted to assist the final selection procedure of virtual screening.

The major strength of this protocol is a comprehensive integration of activity-related factors through machine learning. Comparing with other machine learning studies (9, 10), this research integrated both protein-ligand binding information, ligand-based pharmacophore information and molecular physiochemical properties into an SVM classifier which can yield satisfactory performance without the requirement of a great deal of known inhibitors. Since diverse receptor structures and pharmacophores were considered, more reliable judgments can be made after dimension reduction and model training. Whereas, there still exists some limitations in the screening process. For example, since the virtual screening targeted a specific binding pocket, it was possible that some positive compounds showing different interaction mode would be excluded. It is necessary to further combine virtual screening with different AI methods for improving its prediction ability more accurately.

Experiments showed that this SVM model can effectively exclude over 50% of the inactive compounds in virtual screening and retain the most promising candidates, which can improve the hit rate prominently in the last phase of VS. Moreover, four molecules were successfully predicted using this model, from a set of compounds that were similar to compound **1**. The model displayed better discriminatory power among highly similar candidates.

The identified compounds all bear benzofuran scaffold, which was different from other IRAK1 inhibitors. The acetamide group seems important in maintaining the inhibitory activity of compounds, such as the IC_50_ values of compound **1** vs compound **7**, compound **2** vs compound **9**, and compound **4** vs compound **11**. Moreover, compound **1**, **2,** and **3** were demonstrated an over 40-fold selectivity to IRAK4, showing its potential to be developed as selective IRAK1 inhibitor. The results provide valuable knowledge for further optimization and development of IRAK1 inhibitors and demonstrate that AI can assist VS strategy in a sequential manner for identifying new IRAK1 inhibitors.

## Data Availability Statement

All datasets presented in this study are included in the article/Supplementary Material.

## Author Contributions

JC, RF, JG, and HY processed the data, built the models, analyzed the results, and drafted the manuscript. QW and QH assisted in the design of this study. XD, JW, and BY conceived and designed this research. All authors contributed to the article and approved the submitted version.

## Conflict of Interest

The authors declare that the research was conducted in the absence of any commercial or financial relationships that could be construed as a potential conflict of interest.

## Footnotes

1 www.molport.com
