# Supplementary material for: Evaluation of Artificial Intelligence in Participating Structure-Based Virtual Screening for Identifying Novel Interleukin-1 Receptor Associated Kinase-1 Inhibitors
Source: Front Oncol. 2020 Sep 3;10:1769. doi: 10.3389/fonc.2020.01769 (PMC7494739; doi:10.3389/fonc.2020.01769)
Supplement: Supplementary file 1 [file Data_Sheet_1.docx]

Supplementary Material

# Supplementary Figures and Tables

## Supplementary Figures

**
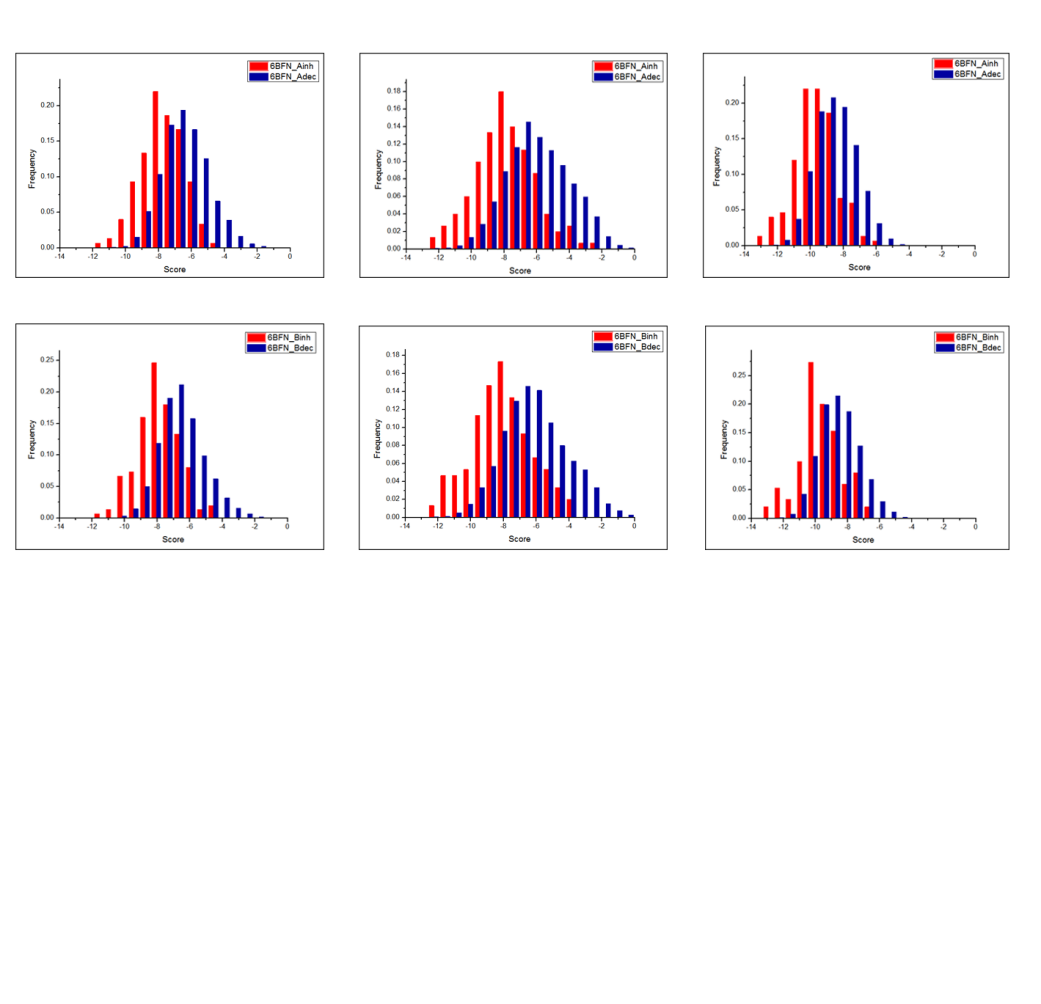
**

**Supplementary Figure S1.** Evaluation of different combination of receptors and docking software.

**
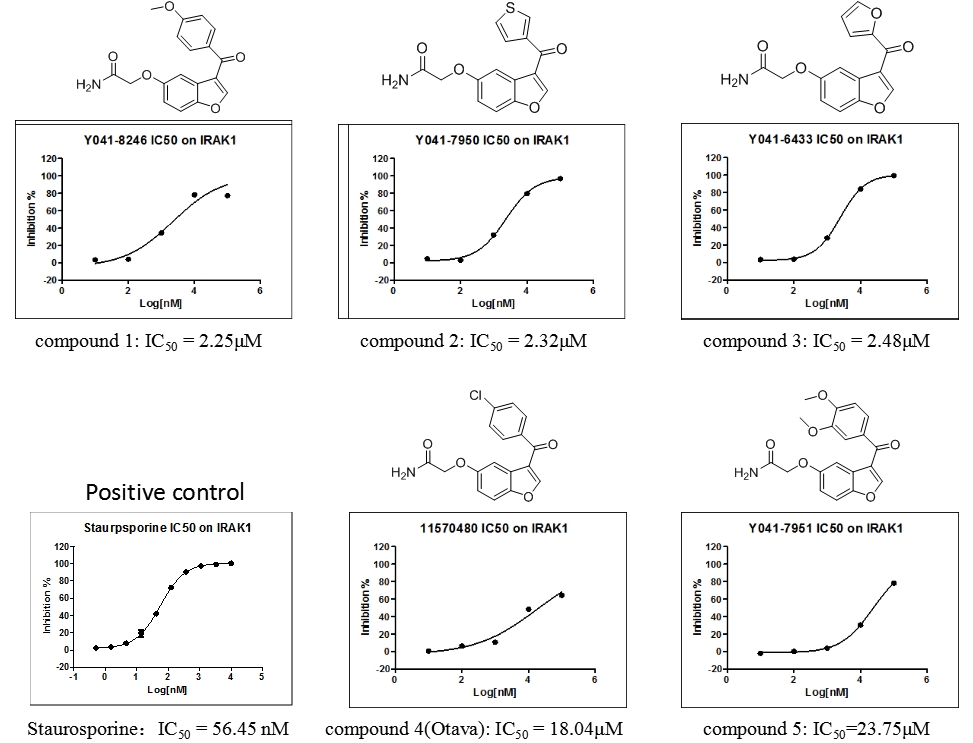
**

**Supplementary Figure S2.** IC_50_ curves of compound 1-5 against IRAK1.


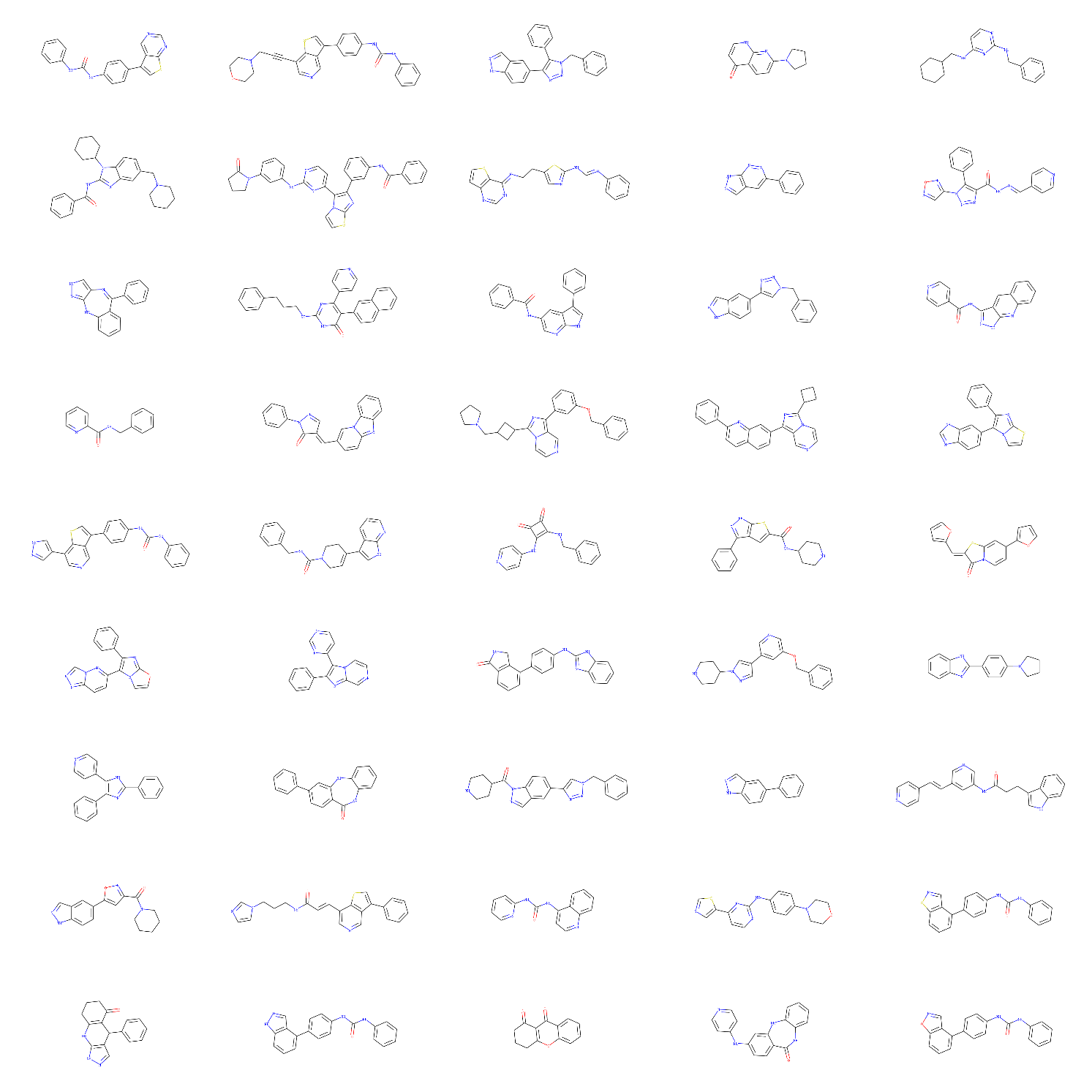


**Supplementary Figure S3.** Structure clustering of IRAK1 inhibitors collected from ChEMBL.


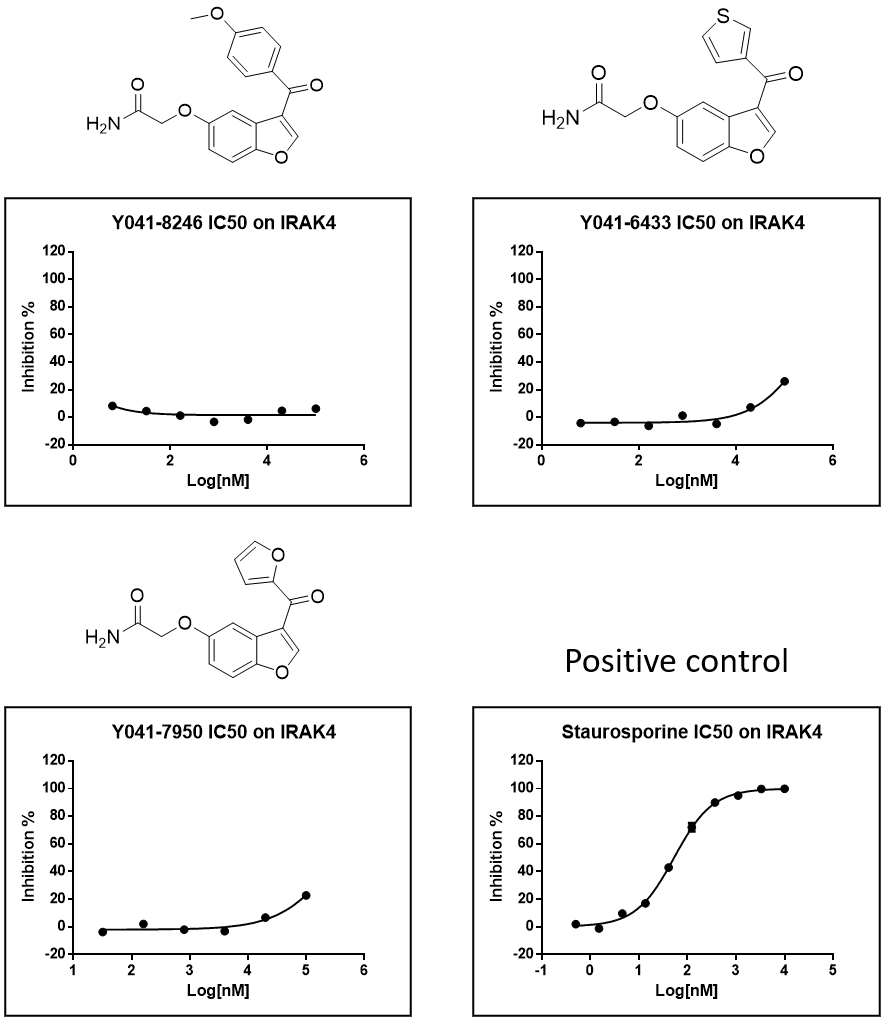


**Supplementary Figure S4.** IC_50_ curves of compound 1-3 against IRAK4.

## Supplementary Tables

**Supplementary Table S1.** 594 IRAK1 inhibitors collected from ChEMBL.

| ChEMBL ID | Smiles |
| --- | --- |
| CHEMBL553 | COCCOc1cc2ncnc(Nc3cccc(c3)C#C)c2cc1OCCOC |
| CHEMBL272453 | COc1ccc(NC(=O)Nc2ccc(cc2)c3cccc4onc(N)c34)cc1Cl |
| CHEMBL1999590 | Cc1cc(c2ccccc2)n(n1)c3cc(NN=Cc4occc4)ncn3 |
| CHEMBL2006276 | COc1cccc2NC(=O)c3ccc(Nc4ccncc4)cc3Nc12 |
| CHEMBL2006493 | FC(F)(F)c1ccc2C(=O)C3=C(CCCC3=O)Oc2c1 |
| CHEMBL1980144 | Cc1ccccc1NC(=O)Nc2ccc(NC(=O)c3csc4ncnc(N)c34)cc2 |
| CHEMBL1970074 | Cc1n[nH]c2cccc(c3ccc(NC(=O)Nc4cccc(c4)C(F)(F)F)cc3)c12 |
| CHEMBL1976420 | CCc1c([nH]c2nccnc12)c3ccc(cc3)C(C)(C)O |
| CHEMBL1997822 | Cc1cc2cc(ccc2[nH]1)c3csc4c(cnc(N)c34)c5cccc(c5)S(=O)(=O)C |
| CHEMBL1988437 | CNC(=O)c1ccccc1Sc2ccc3c(C=Cc4ccccn4)n[nH]c3c2 |
| CHEMBL2005631 | CNC(=O)c1cnc(N)c2c(csc12)c3ccc(NC(=O)Nc4cc(C)ccc4F)cc3 |
| CHEMBL1966703 | Cc1n[nH]c2NC3=C(C(c4ccccc4F)c12)C(=O)CC(C)(C)C3 |
| CHEMBL2000801 | Fc1ccc(NC(=O)Nc2ccc(cc2)c3cccc4sncc34)cc1C(F)(F)F |
| CHEMBL482967 | Cc1nc(N)sc1c2ccnc(Nc3ccc(cc3)N4CCOCC4)n2 |
| CHEMBL1973483 | FC(F)(F)c1ccc2c(NC(=O)Nc3cccc(n3)C(F)(F)F)ccnc2c1 |
| CHEMBL1973211 | Cc1cccc(NC(=O)Nc2ccc(cc2)c3csc4c(cnc(N)c34)C#CCN5CCCC5)c1 |
| CHEMBL1994938 | Nc1ncc(C=CC(=O)NCCCn2ccnc2)c3scc(c4ccc(Br)cc4)c13 |
| CHEMBL1997503 | CC(C)Oc1ccc(cc1Cl)c2noc(n2)c3ccc(NC4CCC(C4)C(=O)O)cc3 |
| CHEMBL1978448 | CS(=O)(=O)N(Cc1ccc2NC(=O)c3cccn3c2c1)C4CC4 |
| CHEMBL1982506 | COc1cc2cnc3C(=O)NC(=O)c3c2cc1OC |
| CHEMBL271381 | COc1ccc(c2ccc(NC(=O)Nc3cccc(Br)c3)cc2)c4c(N)noc14 |
| CHEMBL1997340 | OC1CCCN(C1)C(=O)c2cc(on2)c3ccc4[nH]ncc4c3 |
| CHEMBL583144 | Clc1ccc(Cl)c2c3N=CNC(=O)c3sc12 |
| CHEMBL394619 | Cc1cccc(NC(=O)Nc2ccc(cc2)c3csc4ccnc(N)c34)c1 |
| CHEMBL1983945 | NC(Cc1c[nH]c2ccccc12)C(=O)Nc3cncc(C=Cc4ccncc4)c3 |
| CHEMBL1973893 | CCCS(=O)(=O)Nc1ccc(cc1)c2ccc3[nH]nc(NC(=O)C)c3c2 |
| CHEMBL1978014 | CN1CCC(CC1)C(=O)n2nc(N)c3cc(ccc23)c4cn(Cc5ccccc5)nn4 |
| CHEMBL1973540 | O=C1Nc2ccc3ncsc3c2C1=CNc4ccc(cc4)S(=O)(=O)Nc5ccccn5 |
| CHEMBL1971141 | Cc1cccc(NC(=O)Nc2ccc(cc2)c3cccc(c3)C(=O)N)c1 |
| CHEMBL1967302 | NC(COc1cncc(c1)c2ccc3NC(=O)Cc3c2)Cc4c[nH]c5ccccc45 |
| CHEMBL1995740 | COc1cc(ccc1O)c2ccc3C(=O)Nc4cc(ccc4Nc3c2)[N+](=O)[O-] |
| CHEMBL1969537 | S=C1NN=C2N1C3=NNC(=S)N3c4sc5CCCc5c24 |
| CHEMBL396523 | OC(=O)c1ccccc1Nc2ccnc(Nc3ccc4[nH]ncc4c3)n2 |
| CHEMBL481491 | NC(=O)c1sc(cc1OCc2ccccc2C(F)(F)F)n3cnc4ccccc34 |
| CHEMBL17370 | CSc1ccc(cc1)c2nc(c3ccc(F)cc3)c([nH]2)c4ccncc4 |
| CHEMBL1970203 | Cc1cc(C)cc(NC(=O)Nc2ccc(NC(=O)c3csc4ncnc(N)c34)cc2)c1 |
| CHEMBL1972937 | COc1cccc(C=C2SC(=Nc3ccccc3)NC2=O)c1 |
| CHEMBL1966816 | Fc1cccc2c(NC(=O)Nc3cccc(n3)C(F)(F)F)ccnc12 |
| CHEMBL2000071 | OCCCNc1ncnc2[nH]cc(c3ccccc3)c12 |
| CHEMBL1985074 | CSc1ccc2nc3C(=O)NCCc3c(Cl)c2c1 |
| CHEMBL1969561 | CC(C)N(C)C(=O)c1c(nnn1Cc2ccccc2)c3ccc4[nH]ncc4c3 |
| CHEMBL1995736 | CCOC(=O)c1cccc2[nH]c(nc12)c3ccc(cc3)N4CCC(N)C4 |
| CHEMBL2000832 | COc1cc(ccc1O)c2ccc3C(=O)Nc4cc(ccc4Nc3c2)C(=O)NCc5cccc(F)c5 |
| CHEMBL1971029 | CNS(=O)(=O)c1ccccc1Nc2nc(Nc3cc(ccc3OC)N4CCN(CC4)C(=O)C)ncc2Br |
| CHEMBL1825138 | CC(Oc1cc(cnc1N)c2cnn(c2)C3CCNCC3)c4c(Cl)ccc(F)c4Cl |
| CHEMBL1236126 | CC(C)(C)C(=O)Oc1ccc2c(c1)nc(NC(=O)c3cccc(c3)[N+](=O)[O-])n2CCCO |
| CHEMBL1979057 | O=C1NCc2c1cccc2c3ccc(Nc4nc5ccccc5[nH]4)cc3 |
| CHEMBL1996500 | Cc1nccn2c(c3ccnc(NCC(C)(C)O)n3)c(nc12)c4ccc(F)cc4F |
| CHEMBL1980540 | FC(F)(F)c1cccc(NC(=O)Nc2ccnc3c(Cl)cccc23)n1 |
| CHEMBL1969502 | O=C(Nc1cc2C(=O)NN=Cc3c[nH]c(c1)c23)C4CC4c5ccccc5 |
| CHEMBL1967531 | Nc1ncnc2scc(c3ccc(NC(=O)Nc4ccccc4)cc3)c12 |
| CHEMBL1975233 | COc1cc(C)c(Cl)cc1NC(=O)Nc2cnc(cn2)C#N |
| CHEMBL1967878 | CN1CCN(CC1)c2ccc(Nc3ncc(F)c(NC4C5CC(C=C5)C4C(=O)N)n3)cc2C |
| CHEMBL1972849 | CC(C)(CO)c1nnc2ccc(nn12)c3c(nc4occn34)c5ccc(F)cc5F |
| CHEMBL1682553 | CC(C)Nc1c(nc2ncccn12)c3ccc4[nH]ncc4c3 |
| CHEMBL1972820 | OC(=O)c1ccc(cc1O)N2C(=O)c3ccccc3C2=O |
| CHEMBL1975647 | Fc1cccc2c(NC(=O)Nc3cnccn3)ccnc12 |
| CHEMBL554 | CS(=O)(=O)CCNCc1oc(cc1)c2ccc3ncnc(Nc4ccc(OCc5cccc(F)c5)c(Cl)c4)c3c2 |
| CHEMBL207400 | Cn1nc(c2C(=O)c3cc(Cl)ccc3N(O)c12)C(F)(F)F |
| CHEMBL1996923 | Clc1cc2nn[nH]c2cc1Cl |
| CHEMBL1986943 | OC1CCC(CC1)Nc2nc(Cl)cc(n2)c3c[nH]c4ncccc34 |
| CHEMBL1970340 | Nc1noc2cccc(c3cccc(O)c3)c12 |
| CHEMBL1986869 | Cc1csc(NC(=O)c2sc3nc(ccc3c2N)c4ccncc4)n1 |
| CHEMBL196363 | Cc1cccc(NC(=O)Nc2ccc(cc2)c3csc4ncnc(N)c34)c1 |
| CHEMBL1989805 | NC(=O)C1=C2SC(=Cc3occc3)C(=O)N2C(=C(C#N)C1c4occc4)N |
| CHEMBL1987007 | CN1CCC(CC1)NC(=O)c2cc3c(n[nH]c3s2)c4ccccc4 |
| CHEMBL1997617 | CN1CCN(CC1)C2CCC(CC2)n3cc(c4ccc(Oc5ccccc5)cc4)c6c(N)ncnc36 |
| CHEMBL1973808 | Clc1ccc(cc1)C(=O)Nc2ccc3[nH]ncc3c2 |
| CHEMBL2000354 | COc1ccc(C=C2SC(=Nc3ccccc3)NC2=O)cc1O |
| CHEMBL1982361 | Fc1ccccc1CNC2=C(Nc3ccncc3)C(=O)C2=O |
| CHEMBL1981047 | COC(C(=O)N1Cc2[nH]nc(NC(=O)c3ccc(cc3)N4CCN(C)CC4)c2C1)c5ccccc5 |
| CHEMBL1983534 | C(Nc1nc2ccc(cc2s1)c3ccncc3)c4ccccc4 |
| CHEMBL2006481 | O=C(N1CCCCC1)c2cc(on2)c3ccc4[nH]ncc4c3 |
| CHEMBL1977681 | CNC(=O)c1cc2c(n[nH]c2s1)c3ccccc3F |
| CHEMBL1994241 | COc1cc(C=C(C#N)c2nc3ccccc3[nH]2)c(Br)cc1O |
| CHEMBL1997597 | CCS(=O)(=O)Nc1ccc(cc1)c2ccc3[nH]nc(N)c3c2 |
| CHEMBL1965845 | COc1cccc(c1)C(C)NC(=O)N2CCC(=CC2)c3c[nH]c4ncccc34 |
| CHEMBL2005375 | CCC1N(C2CCCC2)c3nc(Nc4ccc(cc4OC)C(=O)NC5CCCCC5)ncc3N(C)C1=O |
| CHEMBL1999811 | Oc1ccc(C=NNC(=O)Nc2cccc3nsnc23)c(O)c1 |
| CHEMBL1993781 | NC(COCc1ccccc1)COc2cncc(C=Cc3ccncc3)c2 |
| CHEMBL2001646 | Cc1ccccc1c2cccc3c2c(Nc4ccccc4)c(C(=O)O)n3CCCOc5cccc6ccccc56 |
| CHEMBL1992306 | CNC(=O)c1cc(Oc2ccc(NC(=O)Nc3cccc(c3)C(F)(F)F)cc2)ccn1 |
| CHEMBL402846 | Cc1ccc(F)c(NC(=O)Nc2ccc(cc2)c3cncc4c3c(N)nn4C)c1 |
| CHEMBL210032 | CC(=O)Nc1cc(N)c(C#N)c(n1)c2ccccc2 |
| CHEMBL1991285 | Cc1cc(OCC2CCC(C2)C(=O)O)c(NC(=O)Nc3cnc(cn3)C#N)cc1Cl |
| CHEMBL1992922 | OCC(CO)Nc1ncnc2[nH]cc(c3ccccc3)c12 |
| CHEMBL220057 | Cc1cccc(NC(=O)Nc2ccc(cc2)c3cccc4[nH]nc(N)c34)c1 |
| CHEMBL1982711 | NC(=O)c1ccc(cc1)c2c[nH]c3nccc(Cl)c23 |
| CHEMBL491758 | Fc1cnc(Nc2ccccc2)nc1Nc3ccccc3 |
| CHEMBL1993584 | OC(COc1cncc(C=Cc2ccncc2)c1)Cc3c[nH]c4ccccc34 |
| CHEMBL2000894 | Cc1cc(O)ccc1N2C=C(C(=O)NN)C(=O)c3ccc(cc23)c4ccncc4 |
| CHEMBL1984039 | Cc1nn(C)c(C)c1c2cccc3c(CCCOc4cccc5ccccc45)c([nH]c23)C(=O)O |
| CHEMBL1996791 | Nc1ncnc2c1c(I)nn2C3CCC(O)CC3 |
| CHEMBL1983449 | Cc1ccc(CNC2=C(Nc3ccncc3)C(=O)C2=O)cc1C |
| CHEMBL1970142 | CNC(=O)C(C)n1cc(cn1)c2cnc(N)c3c(csc23)c4ccc(NC(=O)Nc5ccc(C)cc5)cc4 |
| CHEMBL1966722 | OC(C(=O)NNC(=S)Nc1ccc(Cl)cc1)(c2ccccc2)c3ccccc3 |
| CHEMBL2002373 | OCCCn1cnc(c2ccccc2)c1c3ccncc3 |
| CHEMBL1988838 | Cc1cnc(Nc2cccc(c2)S(=O)(=O)N)nc1Nc3ccc(OCC(=O)N)cc3 |
| CHEMBL2001288 | Nc1ncnc2c1c(I)nn2C3OC(CO)C(O)C3O |
| CHEMBL1965631 | CN(C)CC(=O)Nc1n[nH]c2ccc(cc12)c3cn(Cc4ccccc4)nn3 |
| CHEMBL1988163 | NS(=O)(=O)c1cccc(Nc2ncc3ccn(c4ccccc4)c3n2)c1 |
| CHEMBL2004419 | CC(C)Cn1c(N)nc2c(F)cc(cc12)c3c(nc4sccn34)c5ccc(F)cc5 |
| CHEMBL1972568 | COc1cncc(C=Cc2ccncc2)c1 |
| CHEMBL1984274 | O=C1NNC(=O)c2c(NN=C3C(=O)N(N=C3c4ccccc4)c5ccccc5)cccc12 |
| CHEMBL1996234 | CC1(O)CC(C1)c2nc(c3ccc4ccc(nc4c3)c5ccccc5)c6c(N)nccn26 |
| CHEMBL1972221 | CCCS(=O)(=O)Nc1ccc(cc1)c2ccc3[nH]nc(NC(=O)CC)c3c2 |
| CHEMBL1972152 | O=C1C2=C(CSC2)Nc3cc(nn13)c4occc4 |
| CHEMBL1979318 | Cc1n[nH]c2OC(=C(C#N)C(c3ccccc3I)c12)N |
| CHEMBL1999931 | O=C(Nc1n[nH]c2ccc(cc12)c3cn(Cc4ccccc4)nn3)c5ccccc5 |
| CHEMBL1971694 | CCCCNC1=NS(=O)N=C1Nc2ccc(F)cc2 |
| CHEMBL1971186 | Fc1ccc(cc1)c2nc3occn3c2c4ccnc(NC5CCNCC5)n4 |
| CHEMBL1998435 | OCCNc1cc2cc(ccc2cn1)c3ccncc3 |
| CHEMBL1991138 | Nc1nccn2c(nc(c3cccc(OCc4ccccc4)c3)c12)C5CC(CN6CCCC6)C5 |
| CHEMBL220241 | CCn1c(nc2cnc(Oc3cccc(NC(=O)c4ccc(OCCN5CCOCC5)cc4)c3)cc12)c6nonc6N |
| CHEMBL1969588 | Clc1cc(cc(NCc2ccccc2)n1)c3c[nH]c4ncccc34 |
| CHEMBL1999126 | CC1=NN(C(=O)C1=Cc2c(C)c(C#N)c3nc4ccccc4n3c2O)c5ccccc5 |
| CHEMBL1969372 | COCCNc1nccc(n1)c2c(nc3cnccn23)c4ccc(F)cc4 |
| CHEMBL1975121 | COc1cccc(c1)C(C)NC(=O)c2ccc(cc2)c3ccncc3 |
| CHEMBL1979883 | CN(C)CC#Cc1cnc(N)c2c(csc12)c3ccc(NC(=O)Nc4cccc(C)c4)cc3 |
| CHEMBL1980489 | Clc1ccc(CNC2=C(Nc3ccncc3)C(=O)C2=O)cc1 |
| CHEMBL2006785 | Cc1ccc(cc1)C(=O)Nc2c(C#N)sc3ccc(Cl)c(Cl)c23 |
| CHEMBL1984711 | COc1cc(Nc2nccc(Nc3onc(c3)C4CCCCC4)n2)cc(OC)c1OC |
| CHEMBL1969126 | CC(C)n1nnc2ccc(cc12)c3c(nc4occn34)c5ccc(F)cc5F |
| CHEMBL1991188 | COc1cccc(c1)C(C)NC(=O)c2ccc(cc2OC)c3ccncc3F |
| CHEMBL1970522 | COc1cc(C=C2SC(=Nc3ccccc3)NC2=O)ccc1O |
| CHEMBL422897 | CN1CCC(C(O)C1)c2c(O)cc(O)c3C(=O)C=C(Oc23)c4ccccc4Cl |
| CHEMBL1981720 | O=C(Nc1cnccn1)Nc2ccnc3ccccc23 |
| CHEMBL2001668 | IC1=CNC(=O)n2cc(nc12)c3ccccc3 |
| CHEMBL1974254 | CCOc1ccc(NC(=O)Nc2ccc(cc2)c3csc4c(cnc(N)c34)c5cnn(C)c5)cc1 |
| CHEMBL1995172 | Brc1ccc2Cc3cn[nH]c3c2c1 |
| CHEMBL316264 | Fc1ccc(cc1)c2nc(c3ccc(F)cc3)c([nH]2)c4ccncc4 |
| CHEMBL2006237 | CN1CCN(CCCNc2cccc(n2)c3nc4c(cccc4[nH]3)C(=O)N)CC1 |
| CHEMBL1990904 | Fc1ccc(cc1)c2nc3cnccn3c2c4ccnc(NCC5CC5)n4 |
| CHEMBL1998112 | COc1ccc(cc1OC)c2cc3nccn3c(Nc4ccccc4C(=O)N)n2 |
| CHEMBL1996831 | OC(=O)c1c2CCC(=C3CCCC3)c2nc4ccccc14 |
| CHEMBL1969506 | CC(C)(C)OC(=O)n1ncc2cc(NC3=C(NCc4ccc(cc4)S(=O)(=O)N)C(=O)C3=O)ccc12 |
| CHEMBL539474 | O=C1CCCN1Cc2ccc3c(NC(=O)c4cccn34)c2 |
| CHEMBL1968590 | CNC(=O)C=Cc1cnc(N)c2c(csc12)c3ccc4sc(C)nc4c3 |
| CHEMBL1969664 | Oc1[nH]c2ccc(cc2c1c3ccc(CN4CCOCC4)cn3)C#N |
| CHEMBL1981079 | NC(=O)c1cc([nH]c1c2ccc(Cl)cc2F)c3ccnc(N)n3 |
| CHEMBL2002446 | CN1CCN(Cc2ccc3c(Cc4c(n[nH]c34)c5csc(c5)C#CCOCC6CC6)c2)CC1 |
| CHEMBL226471 | Fc1ccccc1c2cc(ccn2)c3cc4C(=O)NCCc4[nH]3 |
| CHEMBL1984363 | Cc1cc(NC(=O)Cc2ccc(cc2)c3cccc4[nH]nc(N)c34)ccc1F |
| CHEMBL1990254 | NCC(NC(=O)c1ccc(cc1)c2ccncc2)c3ccccc3 |
| CHEMBL1969843 | C(C1CCOCC1)n2cc(nn2)c3ccc4[nH]ncc4c3 |
| CHEMBL1958401 | O=C1NC(=NC(=C1)c2ccncc2)NC3CCCCC3 |
| CHEMBL1996587 | CC(C)(CO)c1nnc2ccc(nn12)c3c(nc4CCCn34)c5ccc(F)cc5F |
| CHEMBL1967116 | Cc1cccc(NC(=O)Nc2ccc(cc2)c3csc4c(cnc(N)c34)c5cnn(C)c5)c1 |
| CHEMBL1988075 | Cc1ccc(cn1)c2cnc(N)c3c(csc23)c4ccc(NC(=O)Nc5cccc(F)c5)cc4 |
| CHEMBL2005936 | CCCC(=O)Nc1n[nH]c2ccc(cc12)c3nnn(Cc4ccccc4)c3c5ccccc5 |
| CHEMBL1336 | CNC(=O)c1cc(Oc2ccc(NC(=O)Nc3ccc(Cl)c(c3)C(F)(F)F)cc2)ccn1 |
| CHEMBL1998121 | NCCC(C(=O)Nc1ccc2[nH]ncc2c1)c3ccc(Cl)c(Cl)c3 |
| CHEMBL10 | C[S+]([O-])c1ccc(cc1)c2nc(c3ccc(F)cc3)c([nH]2)c4ccncc4 |
| CHEMBL249282 | N#Cc1ccc(cn1)c2n[nH]c3c2Cc4ccc(OCCN5CCOCC5)cc34 |
| CHEMBL1997349 | CC1=NN(C(=O)C1=Cc2c(C)c(C#N)c3nc4ccccc4n3c2O)c5ccc(Cl)c(Cl)c5 |
| CHEMBL2006188 | COc1cccc(c1)C(C)NC(=O)c2ccc(cc2NCCCN)c3ccncc3 |
| CHEMBL514499 | COc1cc2ncn(c3cc(OCc4ccccc4C(F)(F)F)c(s3)C(=O)N)c2cc1OC |
| CHEMBL2003456 | O=C1Nc2cc(cnc2N1)c3ccccc3 |
| CHEMBL1982992 | Oc1cccc(Nc2ncnc3scc(Cl)c23)c1 |
| CHEMBL1996111 | CN(C)C(=O)c1c(nnn1Cc2ccccc2)c3ccc4[nH]ncc4c3 |
| CHEMBL101253 | Clc1ccc(Nc2nnc(Cc3ccncc3)c4ccccc24)cc1 |
| CHEMBL1983195 | CCN(CC)C(=O)Nc1ccc2nc(c3occc3)c(nc2c1)c4occc4 |
| CHEMBL225519 | O=C1NCCc2[nH]c(cc12)c3ccncc3 |
| CHEMBL377408 | CCOc1nc(cc(N)c1C#N)C(=O)NCc2ccccc2S(=O)(=O)N |
| CHEMBL1972584 | CC(=O)N1CCN(CC1)C2CCC(CC2)n3nc(c4ccc(NC(=O)c5cc6ccccc6n5C)cc4)c7c(N)ncnc37 |
| CHEMBL210928 | CCOc1nc(NC(=O)Cc2cc(OC)c(cc2OC)S(=O)(=O)C)cc(N)c1Cl |
| CHEMBL412142 | Cc1c[nH]c(n1)c2cnc(NCCNc3ccc(cn3)C#N)nc2c4ccc(Cl)cc4Cl |
| CHEMBL1682545 | N(c1ccccc1)c2nc(cs2)c3ccc4[nH]ncc4c3 |
| CHEMBL398951 | N#Cc1ccc(cn1)c2n[nH]c3c2Cc4ccc(OCCCCN5CCOCC5)cc34 |
| CHEMBL1997023 | CN(C)c1cc2sncc2cc1NC(=O)C(=O)O |
| CHEMBL2001547 | CCc1nccn2c(c3ccnc(NCC(C)(C)O)n3)c(nc12)c4ccc(F)cc4F |
| CHEMBL1998611 | NC(=O)c1sc2c(Br)ccc(Cl)c2c1N |
| CHEMBL1985092 | Oc1ccc(cc1)c2n[nH]c(Nc3cccc(Cl)c3)c2c4ccc(O)cc4 |
| CHEMBL1984044 | CCN(CC)CCOc1ccc(cc1)c2nc3c(cccc3[nH]2)C(=O)N |
| CHEMBL1990415 | O=C(Nc1n[nH]c2nc3ccccc3cc12)c4ccncc4 |
| CHEMBL231209 | Nc1nccc2scc(c3ccc(NC(=O)Nc4cccc(F)c4)cc3)c12 |
| CHEMBL592030 | NCCCn1cc(c2cc(c3cc4ccccc4s3)c5[nH]ncc5c2)c6nc(N)ncc16 |
| CHEMBL2004447 | CC(C)(C)c1n[nH]c2OC(=C(C#N)C(c3ccccc3F)c12)N |
| CHEMBL1964937 | OCC(NC(=O)N1CCC(=CC1)c2c[nH]c3ncccc23)c4ccccc4 |
| CHEMBL1976134 | c1cc2cc(cnc2[nH]1)c3ccc4cn[nH]c4c3 |
| CHEMBL1977713 | Clc1ccc(CNC(=O)Nc2cccc3[nH]ncc23)cc1 |
| CHEMBL1972489 | Clc1ccc(cc1)C(=O)Nc2ccc3cn[nH]c3c2 |
| CHEMBL1982135 | Cc1ccccc1NC(=O)Nc2ccc3c(CCc4sc5ncnc(N)c5c34)c2 |
| CHEMBL2006580 | Oc1ccc(Nc2ncnc3scc(Cl)c23)cc1 |
| CHEMBL379835 | Cc1nn(c2ccccc2)c3N(O)c4ccc(Cl)cc4C(=O)c13 |
| CHEMBL1971149 | Cc1nccn2c(c3ccnc(NCC(C)(C)C(=O)O)n3)c(nc12)c4ccc(F)cc4F |
| CHEMBL1965570 | Nc1ncc(c2cocc2)c3scc(c4ccc(NC(=O)Nc5ccccc5F)cc4)c13 |
| CHEMBL484390 | CCCCn1c(NC(=O)c2ccc(cc2)C#N)nc3cc(ccc13)N(C)C(=O)C4CCCCC4 |
| CHEMBL259922 | Cc1ccc(cc1Nc2ncnc3c2cnn3c4ccccc4)C(=O)Nc5ccon5 |
| CHEMBL1969879 | CC1CCN(CC1N(C)c2ncnc3[nH]ccc23)C(=O)CO |
| CHEMBL1971649 | Cc1ccc(F)c(NC(=O)Nc2ccc(cc2)c3ccc4nccnc4c3N)c1 |
| CHEMBL1988608 | CC(=O)Nc1cccc(CNC2=C(Nc3ccc4[nH]ncc4c3)C(=O)C2=O)c1 |
| CHEMBL1975128 | Nc1n[nH]c2ccc(cc12)c3nnn(Cc4ccccc4)c3I |
| CHEMBL1682552 | C1CCC(CC1)Nc2c(nc3ncccn23)c4ccc5[nH]ncc5c4 |
| CHEMBL1974416 | Fc1cccc(C(=O)Nc2cnc3[nH]cc(c4ccccc4)c3c2)c1F |
| CHEMBL2003286 | COc1cc(C=C2SC(=O)NC2=O)ccc1O |
| CHEMBL2002649 | COc1ccc2NC(=O)C(=Cc3c[nH]cn3)c2c1 |
| CHEMBL1995813 | NC1CCCCC1Nc2nccc(n2)c3c[nH]c4ncccc34 |
| CHEMBL2006439 | Clc1cc(nc(NC2CCCCC2)n1)c3c[nH]c4ncccc34 |
| CHEMBL1964692 | Cn1nc(N)c2c(cncc12)c3ccc(NC(=O)Nc4cccc(c4)C(F)(F)F)cc3 |
| CHEMBL1970806 | O=C1Nc2sc3CCCCc3c2c4nc(nn14)c5cccnc5 |
| CHEMBL2007064 | Cc1[nH]c(C=C2C(=O)Nc3ccccc23)c(C)c1CCC(=O)O |
| CHEMBL2002702 | Cc1cc(C)c(C=C2C(=O)Nc3ccccc23)[nH]1 |
| CHEMBL2004513 | COC(=O)c1ccccc1c2ccc3C(=O)Nc4ccccc4Nc3c2 |
| CHEMBL1974310 | NC(=O)c1cc2c(cncc2s1)c3ccc(F)cc3 |
| CHEMBL1967887 | Cc1n[nH]c2ccc(cc12)c3cncc(OCCN)c3 |
| CHEMBL1984367 | CN(C)c1ccc(CNC(=O)c2cc3c(n[nH]c3s2)c4ccccc4)cc1 |
| CHEMBL1973937 | Cc1ccc(c2ccc(NC(=O)Nc3cccc(Br)c3)cc2)c4c(N)n[nH]c14 |
| CHEMBL1987679 | NC(COc1cncc(c1)c2ccc3NC(=O)Oc3c2)Cc4c[nH]c5ccccc45 |
| CHEMBL2004290 | COc1ccc(NC(=O)Nc2ccc(cc2)c3csc4c(cnc(N)c34)c5cnn(CC(C)(C)O)c5)cc1 |
| CHEMBL1994864 | Cc1ccc(CNC2=C(Nc3ccncc3)C(=O)C2=O)cc1 |
| CHEMBL2007296 | CCCOc1cccc(c1)C(C)NC(=O)c2ccc(cc2)c3ccncc3 |
| CHEMBL941 | CN1CCN(Cc2ccc(cc2)C(=O)Nc3ccc(C)c(Nc4nccc(n4)c5cccnc5)c3)CC1 |
| CHEMBL183844 | Cc1cccc(NC(=O)Nc2ccc(cc2C)c3cccc4C(=O)NCc34)c1 |
| CHEMBL1973145 | Cc1cccc(NC(=O)Nc2ccc(cc2)c3csc4nc(N)nc(N)c34)c1 |
| CHEMBL1999112 | CCOC(=O)C1=C2SC(=Cc3occc3)C(=O)N2C(=C(C#N)C1c4occc4)N |
| CHEMBL1965423 | CC(C)Cn1c(N)nc2ccc(cc12)c3c(nc4sccn34)c5ccc(F)cc5 |
| CHEMBL1967564 | CCCC(=O)Nc1n[nH]c2nc3ccccc3cc12 |
| CHEMBL2003482 | Clc1ccc(CC(=O)Nc2[nH]nc3CCCc23)cc1 |
| CHEMBL2005828 | Cc1ccc(NC(=O)Nc2ccc(cc2)c3coc4ncnc(N)c34)cc1 |
| CHEMBL1989708 | CC(O)Cn1cc(cn1)c2cnc(N)c3c(csc23)c4ccc(NC(=O)Nc5cccc(C)c5)cc4 |
| CHEMBL1980297 |  |
| CHEMBL1966808 | O=C1Nc2sc3CCCCc3c2c4nc(nn14)c5ccncc5 |
| CHEMBL1970903 | Cc1n[nH]c2ccc(cc12)c3cncc(OCC(N)Cc4c[nH]c5ccccc45)c3 |
| CHEMBL2006631 | CN(C)S(=O)(=O)c1ccccc1c2ccc3C(=O)Nc4ccccc4Nc3c2 |
| CHEMBL2007002 | COc1cc(ccc1O)c2ccc3[nH]nc(C(=O)Nc4ccccc4)c3c2 |
| CHEMBL2004872 | COc1cccc(c1)C(C)NC(=O)c2ccc(cc2)c3ccncn3 |
| CHEMBL1990496 | CN1C(=NC(=C(C1=O)c2ccc3ccccc3c2)c4ccncc4)NCC(N)Cc5ccccc5 |
| CHEMBL248757 | Clc1cc2NC(=O)Nc3cnc(C#N)c(OCCCCOc2cc1NCc4cncs4)n3 |
| CHEMBL1975534 | NCC(O)C(O)Cn1cc(I)c2c(N)ncnc12 |
| CHEMBL1998585 | Nc1ncnc2scc(c3ccc(NC(=O)Nc4cc(ccc4F)C(F)(F)F)cc3)c12 |
| CHEMBL1994693 | Nc1ncnc2sc3CCc4cc(NC(=O)Nc5ccccc5)ccc4c3c12 |
| CHEMBL2002682 | Cc1cccc(Cl)c1c2cccc3c(CCCOc4cccc5ccccc45)c([nH]c23)C(=O)NCCOCCOCCN |
| CHEMBL2005112 | CC1=NN(C(=O)C1=Cc2c(C)c(C#N)c3nc4ccccc4n3c2O)c5cccc(Br)c5 |
| CHEMBL1999496 | Cc1cccc(c1)C2(CC2C(=O)Nc3ccncc3)c4cccc(C)c4 |
| CHEMBL1966143 | CC1=NN(C(=O)C1=Cc2c(C)cc3nc4ccccc4n3c2O)c5ccc(cc5)C(=O)O |
| CHEMBL1084546 | CN(c1ncccc1CNc2nc(Nc3ccc4NC(=O)Cc4c3)ncc2C(F)(F)F)S(=O)(=O)C |
| CHEMBL1991356 | COc1cccc(c1)c2cc3C(=O)Nc4ccccc4n3c2 |
| CHEMBL1980391 | COc1cc2Nc3n[nH]c(C)c3N=C(c4ccccc4Cl)c2cc1F |
| CHEMBL1982924 | COc1cccc(c1)C(=O)Nc2cnc3[nH]cc(c4ccccc4)c3c2 |
| CHEMBL519697 | CN(C(=O)C1CCCCC1)c2ccc3c(c2)nc(NC(=O)c4ccc(cc4)C#N)n3C |
| CHEMBL1965836 | Cc1n[nH]c2ccc(cc12)c3cncc(OCC(N)Cc4cccc(OC(F)(F)F)c4)c3 |
| CHEMBL1991143 | Cc1ccc(cc1)n2nc(C)c3C(=O)c4cc(Cl)ccc4N(O)c23 |
| CHEMBL1997534 | COc1cccc(c1)C(C)NC(=O)c2cc(C)c(s2)c3ccc4[nH]nc(C)c4c3 |
| CHEMBL52387 | CCC(CO)Nc1nc(NCc2ccccc2)c3ncn(C(C)C)c3n1 |
| CHEMBL1982563 | COCC(C)(C)CNc1nccc(n1)c2c(nc3c(C)nccn23)c4ccc(F)cc4 |
| CHEMBL1966628 | NC1CCC(CC1)Nc2nccc(n2)c3c[nH]c4ncccc34 |
| CHEMBL1991674 | OC1CCC(CC1)Nc2cc(Cl)nc(n2)c3c[nH]c4ncccc34 |
| CHEMBL1987793 | Cc1ccccc1c2c(C(=O)O)n(CCCOc3cccc4ccccc34)c5ccccc25 |
| CHEMBL1968380 | CCC1N(C2CCCC2)c3nc(Nc4ccc(cc4OC)C(=O)NC5CCN(C)CC5)ncc3N(C)C1=O |
| CHEMBL2003768 | COc1ccc(CNC2=C(Nc3ccncc3)C(=O)C2=O)c(OC)c1 |
| CHEMBL1980163 | C(Oc1ccc(Nc2ncnc3ccccc23)cc1)c4ccccc4 |
| CHEMBL1964290 | Cc1cccc(c1C)c2[nH]c(cc2C(=O)N)c3ccnc(N)n3 |
| CHEMBL1977374 | CC(C)c1nnc2ccc(nn12)c3c(nc4CCCn34)c5ccc(F)cc5F |
| CHEMBL1419458 | Nc1nonc1n2nnc(C(=O)NN=Cc3ccncc3)c2c4ccccc4 |
| CHEMBL1979093 | COc1ccc(c2ccc(NC(=O)Nc3ccc(F)c(C)c3)cc2)c4c(N)noc14 |
| CHEMBL1967094 | Clc1ccc(CNC(=O)c2cc3c(n[nH]c3s2)c4ccccc4)cc1 |
| CHEMBL1969301 | CCCCn1c(NC(=O)c2cccc(c2)C#N)nc3cc(ccc13)N(C)C(=O)C4CCCCC4 |
| CHEMBL1980704 | Cc1cc2nnc(SCc3cn4ccccc4n3)n2c5ccccc15 |
| CHEMBL1982660 | COc1ccc2c(NC(=O)Nc3cccc(n3)C(F)(F)F)ccnc2c1 |
| CHEMBL1992555 | [O-][N+](=O)c1cccc(c1)C(=O)NN=Cc2oc(Sc3nc4ccccc4[nH]3)cc2 |
| CHEMBL1977135 | O=C(NCc1ccccc1)c2ccc(cc2)c3ccncc3 |
| CHEMBL1516890 | Cn1cc(C=C2C(=O)Nc3cccnc23)c4ccccc14 |
| CHEMBL260135 | Nc1n[nH]c2nnc(c3ccccc3)c(c4ccccc4)c12 |
| CHEMBL497151 | Ic1ccc(Oc2cncc3sc(cc23)C4=NC(=O)ON4)cc1 |
| CHEMBL1987910 | Nc1nccc2scc(c3ccc(NC(=O)Nc4ccccc4C(F)(F)F)cc3)c12 |
| CHEMBL1966035 | CC(NC1=C(Nc2ccncc2)C(=O)C1=O)c3ccccc3 |
| CHEMBL378627 | COc1cc(c(OC)cc1CC(=O)Nc2cc(N)c(C#N)c(OC(C)C)n2)S(=O)(=O)C |
| CHEMBL2005886 | Cc1cc(cc2[nH]c(nc12)C3=C(NCC(O)c4cccc(Cl)c4)C=CNC3=O)N5CCOCC5 |
| CHEMBL1989029 | CC(C)OC1=NNC(=O)C1=Cc2c[nH]c3ccccc23 |
| CHEMBL1964687 | Cc1cc(C)cc(NC(=O)Nc2ccc3c(CCc4sc5ncnc(N)c5c34)c2)c1 |
| CHEMBL223460 | CS(=O)(=O)Nc1cc2OCCCCCOc3nc(NC(=O)Nc2cc1Cl)cnc3C#N |
| CHEMBL184847 | Cc1ccc(NC(=O)Nc2ccc(cc2)c3cccc4C(=O)NCc34)cc1C |
| CHEMBL2000345 | COc1cc(C=C(C#N)c2nc3cc(C)ccc3[nH]2)c(Br)cc1O |
| CHEMBL1682558 | Fc1ccc(cc1)c2ncn(CCN3CCOCC3)c2c4ccc5[nH]ncc5c4 |
| CHEMBL2005475 | O=C(NCC1CCCCC1)c2cc3c(n[nH]c3s2)c4ccccc4 |
| CHEMBL2002726 | Cc1n[nH]c2ccc(cc12)c3cncc(OCC(N)Cc4ccccc4)c3 |
| CHEMBL1983963 | Cc1cccc(NC(=O)Nc2ccc(cc2)c3coc4ncnc(N)c34)c1 |
| CHEMBL1991434 | COc1ccc(C=C2C(=O)ON=C2c3ccc(Br)cc3)cc1OC |
| CHEMBL1998829 | Clc1ccc(cc1)C(=O)Nc2cnc3[nH]cc(c4ccccc4)c3c2 |
| CHEMBL1421 | Cc1nc(Nc2ncc(s2)C(=O)Nc3c(C)cccc3Cl)cc(n1)N4CCN(CCO)CC4 |
| CHEMBL1975256 | Nc1nonc1n2nnc(C(=O)NN=Cc3cccs3)c2c4cccs4 |
| CHEMBL1974935 | NC(COc1cncc(C=Cc2ccncc2)c1)Cc3c[nH]c4ccccc34 |
| CHEMBL1989265 | CC1CSC2=C(C(=O)O)C(=O)c3cc(F)c(cc3N12)N4CCC(N)C4 |
| CHEMBL306380 | Cc1ccc(cc1)c2nn(c3ncnc(N)c23)C(C)(C)C |
| CHEMBL1969049 | COc1cccc(c1)C(C)NC(=O)c2ccc(c(C)c2)c3ccncc3 |
| CHEMBL1998545 | OCC(Cc1ccccc1)NC(=O)c2ccc(cc2)c3ccncc3 |
| CHEMBL1998159 | Cc1n[nH]c2cnc(cc12)c3cncc(OCC(N)Cc4cccc(c4)C(F)(F)F)c3 |
| CHEMBL2000408 | Cc1cn2c(c3ccnc(NCC(C)(C)CO)n3)c(nc2c(C)n1)c4ccc(F)cc4F |
| CHEMBL1972258 | NCCCOc1cncc(C=Cc2ccncc2)c1 |
| CHEMBL1966087 | C1CC(CN1)Oc2cncc(C=Cc3ccncc3)c2 |
| CHEMBL1998414 | Cc1n[nH]c2sc(cc12)C(=O)NCCc3ccc(Cl)cc3 |
| CHEMBL117697 | COc1cccc(Nc2ncnc3ccccc23)c1 |
| CHEMBL1974803 | OC1=C(c2cc(Cl)ccc2O)c3c(Cl)cc(Cl)cc3NC1=O |
| CHEMBL1994538 | FC(F)(F)c1cccc2c(NC(=O)Nc3cnccn3)ccnc12 |
| CHEMBL185569 | Cc1cccc(NC(=O)Nc2ccc(cc2)c3cccc4C(=O)NCc34)c1 |
| CHEMBL211378 | Cc1cccc(NC(=O)Nc2ccc(cc2)c3noc4ncnc(N)c34)c1 |
| CHEMBL1969190 | COc1ccc(c2ccc(NC(=O)Nc3cc(F)cc(F)c3)cc2)c4c(N)noc14 |
| CHEMBL1968791 | Cc1c(c2cccc(OCc3ccccc3)c2)c4c(N)ncnc4n1C5CC(CN6CCCC6)C5 |
| CHEMBL1984700 | CC(C)Oc1nccn2c(c3ccnc(NCC(C)(C)O)n3)c(nc12)c4ccc(F)cc4F |
| CHEMBL1986530 | Fc1ccc(CC2=NNC(=O)C3=C2CCCC3)cc1C(=O)N4CCN(CC4)c5ncccn5 |
| CHEMBL1985095 | NC(=O)Cn1cc(c2cc(c3cc4ccccc4s3)c5[nH]ncc5c2)c6nc(N)ncc16 |
| CHEMBL3735504 | CC(C)(C)C(=O)Nc1ccc2c(c1)nc(NC(=O)c3cccc(c3)C(F)(F)F)n2[C@@H]4CC[C@H](CO)CC4 |
| CHEMBL509032 | COc1cc(ccc1Nc2ncc(Cl)c(Nc3ccccc3S(=O)(=O)C(C)C)n2)N4CCC(CC4)N5CCN(C)CC5 |
| CHEMBL1977223 | CCCC(=O)Nc1n[nH]c2ccc(cc12)c3cn(Cc4ccccc4)nn3 |
| CHEMBL2001224 | Cc1cc(OC2CCCC2)c(NC(=O)Nc3cnc(cn3)C#N)cc1Cl |
| CHEMBL1996066 | Nc1nc2ccc(cc2n1c3nc(cs3)c4ccccc4)C#N |
| CHEMBL1966279 | NCCCNS(=O)(=O)c1cccc(c1)C(=O)Nc2ccc(cc2)C3=NNC(=O)c4ccccc34 |
| CHEMBL208637 | CCOc1nc(NC(=O)C)cc(N)c1C#N |
| CHEMBL2002165 | CC(O)Cn1cc(cn1)c2cnc(N)c3c(csc23)c4ccc(NC(=O)Nc5cccc(F)c5)cc4 |
| CHEMBL1979970 | CCCC(=O)Nc1n[nH]c2nnc(cc12)c3cccc(F)c3F |
| CHEMBL1976220 | Nc1nccc2scc(c3ccc(NC(=O)Nc4cccc(Br)c4)cc3)c12 |
| CHEMBL1984788 | NC(=O)c1cc(Cl)cc2[nH]c(nc12)c3ccc(cc3F)C4CCCNC4 |
| CHEMBL1997846 | Clc1cc(NC2CCCCC2)nc(n1)c3c[nH]c4ncccc34 |
| CHEMBL1977346 | Ic1cc2C(=O)Nc3ccccc3Nc2nn1 |
| CHEMBL1974288 | OCC(NC(=O)c1ccc(cc1)c2ccncc2)c3ccccc3 |
| CHEMBL2006010 | FC(F)(F)c1cccc(NC(=O)Nc2ccnc3c(Cl)c(Cl)ccc23)n1 |
| CHEMBL1991800 | CC(C)(CO)CNc1nccc(n1)c2c(nc3c(CC4CC4)nccn23)c5ccc(F)cc5 |
| CHEMBL362558 | Fc1cc2CN(CCn3cc(C4=C(C(=O)NC4=O)c5cnc6ccccn56)c(c1)c23)C(=O)N7CCCCC7 |
| CHEMBL1989474 | Cn1cc(c2ccncn2)c(n1)c3ccc(F)cc3 |
| CHEMBL1727312 | O=C1Nc2ccccc2Nc3ccccc13 |
| CHEMBL340384 | NC(=O)C1=CN(c2ccc(O)cc2Cl)c3cc(ccc3C1=O)c4ccncc4 |
| CHEMBL2003638 | Fc1cccc(c1)C(=O)Nc2n[nH]c3ccc(cc23)c4cn(Cc5ccccc5)nn4 |
| CHEMBL1999714 | Cc1ccc2Nc3ccccc3C(=O)Nc2c1 |
| CHEMBL1988387 | Cn1cc(cn1)c2cnc(N)c3c(csc23)c4ccc(NC(=O)Nc5ccc(OC(F)F)cc5)cc4 |
| CHEMBL1994526 | CC(N)C1CCC(CC1)C(=O)Nc2ccncc2 |
| CHEMBL1965660 | NC(=O)c1cc([nH]c1c2ccc(Cl)cc2Cl)c3ccnc(N)n3 |
| CHEMBL1987009 | COc1cccc(CNC(=O)c2cc3c(n[nH]c3s2)c4ccccc4)c1 |
| CHEMBL2005186 | CC(C)NC(=O)COc1cccc(c1)c2nc(Nc3ccc4[nH]ncc4c3)c5ccccc5n2 |
| CHEMBL2004716 | CCN(CC)CCNC(=O)C=Cc1cnc(N)c2c(csc12)c3ccc(NC(=O)Nc4cccc(C)c4)cc3 |
| CHEMBL1993548 | CC(N)COc1cnc(Cl)c(C=Cc2ccncc2)c1 |
| CHEMBL1974328 | Cc1cccc(Nc2nc(NC3CCCCC3N)cnc2C(=O)N)c1 |
| CHEMBL475251 | COc1cc(Nc2ncc(F)c(Nc3ccc4OC(C)(C)C(=O)Nc4n3)n2)cc(OC)c1OC |
| CHEMBL2001485 | CC(C(=O)N(C)C)n1cc(cn1)c2cnc(N)c3c(csc23)c4ccc(NC(=O)Nc5ccc(C)cc5)cc4 |
| CHEMBL482767 | OC(=Nc1cccc(Cl)c1)Nc2ncc(CCN=C3NC=Nc4ccsc34)s2 |
| CHEMBL1975357 | CC(C)(C)OC(=O)n1cc(c2cncn2C3CCCCC3)c4ccccc14 |
| CHEMBL2005528 | CCOC(=O)c1ccc2[nH]c3C(=O)NCCc3c2c1 |
| CHEMBL2006715 | Fc1cccc(F)c1C(=O)Nc2cccc(c2)c3nc4sccn4c3c5ccnc(Nc6cccc(c6)N7CCCC7=O)n5 |
| CHEMBL256835 | COc1nccc(n1)c2c(ncn2C3CCC(O)CC3)c4ccc(F)cc4 |
| CHEMBL1375640 | Cc1nc(COc2ccccc2Cl)sc1c3ccnc(N)n3 |
| CHEMBL1966343 | CC(C)Cc1cc(on1)c2ccc3[nH]nc(N)c3c2 |
| CHEMBL535331 | N(N=Cc1c[nH]c2ccccc12)c3nc4ccccc4[nH]3 |
| CHEMBL210618 | CCOc1nc(NC(=O)Cc2cc(OC)ccc2OC)cc(N)c1C#N |
| CHEMBL1964804 | CS(=O)(=O)NCCOc1cncc(c1)c2ccc3cnccc3c2 |
| CHEMBL2001957 | CC(C)(CO)CNc1nccc(n1)c2c(nc3c(OCC(F)(F)F)nccn23)c4ccc(F)cc4 |
| CHEMBL2000335 | Cc1ccc(NC(=O)Nc2ccc(cc2)c3csc4ncnc(N)c34)cc1C |
| CHEMBL1966514 | Nc1ncc(c2cocc2)c3scc(c4ccc(NC(=O)Nc5cccc(F)c5)cc4)c13 |
| CHEMBL243088 | OC(=O)c1ccccc1Nc2ccnc(Nc3cccc(O)c3)n2 |
| CHEMBL1970217 | COc1cccc(c1)C(C)NC(=O)c2ccc(cc2)c3ccncc3C |
| CHEMBL1982383 | CC(C)(CO)CNc1nccc(n1)c2c(nc3c(CCN)nccn23)c4ccc(F)cc4 |
| CHEMBL3736036 | OC[C@@H]1CC[C@@H](CC1)n2c(NC(=O)c3cccc(c3)C(F)(F)F)nc4cc(CN5CCCCC5)ccc24 |
| CHEMBL262433 | Cc1ccc(F)c(NC(=O)Nc2ccc(cc2)c3nsc4ncnc(N)c34)c1 |
| CHEMBL1977148 | COc1ccc(NC(=O)Nc2ccc(cc2)c3csc4c(cnc(N)c34)c5cnn(CCO)c5)cc1 |
| CHEMBL1999428 | CC(NC(=O)c1ccc(cc1)c2ccncc2)c3ccccc3 |
| CHEMBL1972142 | O=C(NCc1ccccc1)c2cccc(c2)c3cnc4[nH]ccc4c3 |
| CHEMBL1972183 | CN1CCCC1COc2cncc(CCc3ccccc3)c2 |
| CHEMBL2004544 | Oc1cccc2c(ccc(NCc3ccc4OCOc4c3)c12)S(=O)(=O)O |
| CHEMBL1970735 | CCCOc1nc(NC(=O)C)cc(N)c1C#N |
| CHEMBL2004365 | CCOC(=O)C1=C(N)N2C(=O)C(=Cc3cccs3)SC2=C(C1c4cccs4)C(=O)N |
| CHEMBL394790 | Nc1nccc2scc(c3ccc(NC(=O)Nc4cccc(Cl)c4)cc3)c12 |
| CHEMBL363648 | CCc1nc(c2cccc(C)c2)c(s1)c3ccnc(NC(=O)c4ccccc4)c3 |
| CHEMBL3736465 | OCCc1ccc(cc1)n2c(NC(=O)c3cccc(c3)C(F)(F)F)nc4cc(CN5CCCCC5)ccc24 |
| CHEMBL443962 | COc1ccc(cc1)c2cnc3c(Br)cnn3c2 |
| CHEMBL371206 | Cc1cc(C)cc(NC(=O)Nc2ccc(cc2)c3c(C)sc4ncnc(N)c34)c1 |
| CHEMBL1980896 | Nc1ncnc2sc(Br)c(c3ccc(NC(=O)Cc4cccc(Cl)c4)cc3)c12 |
| CHEMBL1970369 | NC(=O)C1CCCN(C1)C(=O)c2cc(on2)c3ccc4[nH]ncc4c3 |
| CHEMBL1968406 | CCc1cccc(NC(=O)Nc2ccc3c(CCc4sc5ncnc(N)c5c34)c2)c1 |
| CHEMBL1986603 | Cc1onc(NC(=O)CCNC(=O)Nc2nc(C)c(s2)c3ccc(cc3)n4cccn4)c1 |
| CHEMBL1981725 | CCN1CCN(CC1)c2ccc(Nc3nccc(n3)c4c(nc5ccccn45)c6cccc(c6)C(=O)Nc7ccccc7F)cc2 |
| CHEMBL1982980 | CC(=C(C)c1cccc2c(CCCOc3cccc(Cl)c3Cl)c([nH]c12)C(=O)O)C |
| CHEMBL2001239 | Nc1ncnc(Nc2cc(CNC(=O)C(F)(F)F)c(O)c(c2)c3ccc(Cl)cc3)n1 |
| CHEMBL526133 | O=C1NC(=CC(=N1)c2ccc3[nH]ncc3c2)c4ccccc4 |
| CHEMBL522892 | CN1CCN(CC1)c2ccc3nc([nH]c3c2)C4=C(N)c5c(F)cccc5NC4=O |
| CHEMBL1990635 | CC1CCCN1CCc2cc3cc(ccc3[nH]2)c4ccc(OC(F)(F)F)cc4 |
| CHEMBL2003271 | NC(=O)c1sc2ccc(Cl)c(Cl)c2c1NC(=O)c3ccc(Cl)cc3 |
| CHEMBL1983932 | COc1ccc2c(NC(=O)Nc3cccc(Br)n3)ccnc2c1 |
| CHEMBL3735719 | [O-][N+](=O)c1cccc(c1)C(=O)Nc2nc3cc(CN4CCCCC4)ccc3n2c5ccccc5 |
| CHEMBL1969042 | Oc1ccc(Nc2[nH]nc(c3ccc(O)cc3)c2c4ccc(O)cc4)cc1 |
| CHEMBL1984038 | COc1cc(cc(OC)c1OC)c2ccc3Nc4cc(ccc4C(=O)Nc3c2)c5ccccc5NS(=O)(=O)C |
| CHEMBL1977604 | CC1CSC2=C(C(=O)O)C(=O)c3cc(F)c(N4CCC(N)C4)c(F)c3N12 |
| CHEMBL1992323 | Clc1ccc2oc3C(=O)NC=Nc3c2c1 |
| CHEMBL1995832 | COc1nccn2c(c3ccnc(NCC(C)(C)CO)n3)c(nc12)c4ccc(F)cc4F |
| CHEMBL462120 | NC1=C(c2ccccc2)c3cc(Cl)ccc3NC1=O |
| CHEMBL2000429 | CC(C)Oc1nccn2c(c3ccnc(NCC(C)(C)CO)n3)c(nc12)c4ccc(F)cc4F |
| CHEMBL1375418 | Cc1ccc(CNc2ccc3nnc(c4ccccc4)n3n2)cc1 |
| CHEMBL1968151 | O=C1Nc2sccc2c3nc(nn13)c4ccncc4 |
| CHEMBL1972158 | Nc1n[nH]c2ccc(cc12)c3nnn(Cc4ccccc4)c3c5ccccc5 |
| CHEMBL1975500 | COc1cc(ccc1O)c2ccc3C(=O)Nc4cc(ccc4Nc3c2)C(=O)NCCNC(=O)C |
| CHEMBL1982866 | COCCn1cc(c2ccncn2)c(n1)c3ccc(Cl)cc3 |
| CHEMBL404367 | Cc1cccc(NC(=O)Nc2ccc(cc2)c3cncc4c3c(N)nn4C)c1 |
| CHEMBL1976093 | CN(C)CC(C)(C)CNc1nccc(n1)c2c(nc3cnccn23)c4ccc(F)cc4 |
| CHEMBL1990884 | Cc1cc(C)c2oc(Nc3ccc(cc3F)c4cccc(C(=O)N)c4N)nc2c1 |
| CHEMBL1986970 | COCCN(C)C(=O)c1c(nnn1Cc2ccccc2)c3ccc4[nH]ncc4c3 |
| CHEMBL1965589 | Cc1nccn2c(c3ccnc(NCC4(O)CC4)n3)c(nc12)c5ccc(F)cc5F |
| CHEMBL1984633 | COc1cccc(c1)C(C)NC(=O)c2cnc(s2)c3ccncc3 |
| CHEMBL1972576 | Cc1ccc(NC(=O)Nc2ccc(NC(=O)c3csc4ncnc(N)c34)cc2)cc1 |
| CHEMBL1980142 | FC(F)(F)c1cccc(NC(=O)Nc2ccnc3c(cccc23)C(F)(F)F)n1 |
| CHEMBL1984586 | Cc1ccccc1NC(=O)Nc2ccc(cc2)c3coc4ncnc(N)c34 |
| CHEMBL213505 | Nc1ncnc2onc(c3ccc(NC(=O)Nc4cccc(c4)C(F)(F)F)cc3)c12 |
| CHEMBL1993424 | NS(=O)(=O)c1cccc(Nc2ncc3ccn(Cc4ccccc4)c3n2)c1 |
| CHEMBL2006778 | CNC(=O)C=Cc1cnc(N)c2c(csc12)c3cc(F)c4[nH]c(C)cc4c3 |
| CHEMBL1461728 | Fc1cccc(c1)C(=O)Nc2ccc3[nH]ncc3c2 |
| CHEMBL508928 | Cc1cccc(NC(=O)Nc2ccc(cc2)c3cnc4c(cnn4c3N)c5cnn(C)c5)c1 |
| CHEMBL1682540 | c1cnc2nc(cn2c1)c3ccc4[nH]ncc4c3 |
| CHEMBL1976090 | Nc1ncnc2c1sc3ncnc(N)c23 |
| CHEMBL1999718 | FC(F)(F)c1ccc2c(NC(=O)Nc3ccc(cn3)C#N)ccnc2c1 |
| CHEMBL41783 | CN(C)CCCC(=O)Nc1n[nH]c2nnc(cc12)c3cccc(F)c3F |
| CHEMBL274064 | Nc1nccc(n1)c2c(ncn2C3CCNCC3)c4ccc(F)cc4 |
| CHEMBL1997764 | COc1ccc(cc1)C(=O)Nc2cnc3[nH]cc(c4ccccc4)c3c2 |
| CHEMBL1964644 | [O-][N+](=O)c1cnc(NC(=O)NCc2ccccc2)s1 |
| CHEMBL1990885 | CCN(CC)CCNC(=O)c1c(C)[nH]c(C=C2C(=O)Nc3ccc(F)cc23)c1C |
| CHEMBL1970189 | Cc1n[nH]c2OC(=C(C#N)C(c3ccccc3Cl)c12)N |
| CHEMBL1971943 | Cc1c[nH]c2nccc(Oc3c(F)cc(Nc4cc(Cl)nc(N)n4)cc3F)c12 |
| CHEMBL1975900 | Clc1ccc(CNC2=C(Nc3ccncc3)C(=O)C2=O)cc1Cl |
| CHEMBL1967720 | CC(C)(C(=O)N)n1cc(cn1)c2cnc(N)c3c(csc23)c4ccc(NC(=O)Nc5cccc(F)c5)cc4 |
| CHEMBL1988173 | Cc1cc(Cl)ccc1NC(=S)NNC(=O)C(O)(c2ccccc2)c3ccccc3 |
| CHEMBL1971430 | CN1C(=NC(=C(C1=O)c2cccc(C)c2)c3ccncc3)NCC(N)Cc4ccccc4 |
| CHEMBL483158 | COc1cccc(F)c1C2=NCc3cnc(Nc4ccc(C(=O)O)c(OC)c4)nc3c5ccc(Cl)cc25 |
| CHEMBL1987034 | NS(=O)(=O)c1cccc(Nc2ncc(Br)c(Nc3ccc(OCC#N)cc3)n2)c1 |
| CHEMBL1981782 | Clc1ccc(CNC(=O)Nc2ccc3[nH]ncc3c2)cc1Cl |
| CHEMBL340921 | Cc1cc(O)ccc1N2C=C(C(=O)N)C(=O)c3ccc(cc23)c4ccncc4 |
| CHEMBL1968926 | C(Sc1nc(n[nH]1)c2ccncc2)c3ccccc3 |
| CHEMBL1979357 | Cn1cc(C(CN)c2cncc(C=Cc3ccncc3)c2)c4ccccc14 |
| CHEMBL1978200 | Cc1nccn2c(c3ccnc(NCCC(C)(C)O)n3)c(nc12)c4ccc(F)cc4F |
| CHEMBL1993243 | CNC(=O)C=Cc1cnc(N)c2c(csc12)c3ccc4OCOc4c3 |
| CHEMBL2005899 | CC(NC1=C(N(C(=O)C)c2ccncc2)C(=O)C1=O)C(C)(C)C |
| CHEMBL2000481 | NCCNS(=O)(=O)c1cccc(c1)C(=O)Nc2ccc(cc2)C3=NNC(=O)c4ccccc34 |
| CHEMBL1965507 | Cc1cccc(NC(=O)Nc2ccc(NC(=O)c3csc4ncnc(N)c34)cc2)c1 |
| CHEMBL246970 | NCC1CCC(CNc2nc(NCc3ccccc3Cl)ncc2[N+](=O)[O-])CC1 |
| CHEMBL2002992 | CC(N)C1CCC(CC1)C(=O)Nc2ccnc3[nH]ccc23 |
| CHEMBL1984191 | Clc1ccc(cc1Cl)c2n[nH]cc2c3ccncn3 |
| CHEMBL572878 | CN1CCN(CC1)c2cc(Nc3cc(C)[nH]n3)nc(Sc4ccc(NC(=O)C5CC5)cc4)n2 |
| CHEMBL1971245 | CN1CCN(CC1)c2cccc(Nc3nccc(n3)c4c(nc5ccccn45)C(=O)N)c2 |
| CHEMBL411903 | Nc1ncnc2NCCC(=Nc12)c3ccc(NC(=O)Nc4cc(ccc4F)C(F)(F)F)cc3 |
| CHEMBL1976240 | COc1cccc(c1)C(CN)NC(=O)c2ccc(cc2)c3ccncc3 |
| CHEMBL1970709 | Brc1ccc(CNC(=O)Nc2ccc3[nH]ncc3c2)cc1 |
| CHEMBL1994830 | Nc1oc(nn1)c2cc3c(Oc4ccc(Cl)cc4)cncc3s2 |
| CHEMBL939 | COc1cc2ncnc(Nc3ccc(F)c(Cl)c3)c2cc1OCCCN4CCOCC4 |
| CHEMBL1979176 | COc1ccc2c(NC(=O)Nc3cc(ccn3)C(F)(F)F)ccnc2c1 |
| CHEMBL1974157 | Oc1ccc(C=C(C#N)C(=O)NCc2ccccc2)cc1O |
| CHEMBL223360 | Cc1ccc(F)c(NC(=O)Nc2ccc(cc2)c3cccc4[nH]nc(N)c34)c1 |
| CHEMBL2004871 | OCCCn1cnc(c2ccc(F)cc2)c1c3ccncc3 |
| CHEMBL1987533 | Clc1ccccc1CC(=O)Nc2ccc3cn[nH]c3c2 |
| CHEMBL3734814 | OC[C@@H]1CC[C@@H](CC1)n2c(NC(=O)c3cccc(c3)C(F)(F)F)nc4ccc(CO)cc24 |
| CHEMBL2001920 | Brc1ccc2NC(=O)C(=C3SC(=S)NC3=O)c2c1 |
| CHEMBL1986328 | CN(C)C(=O)Nc1ccc2nc(c3occc3)c(nc2c1)c4occc4 |
| CHEMBL1976455 | FC(F)(F)c1ccc2nccc(NC(=O)Nc3cnccn3)c2c1 |
| CHEMBL1964777 | NC(=O)c1cc2c(Oc3cccc(F)c3)cncc2s1 |
| CHEMBL1983309 | COc1ccc(C(=O)Nc2nc3ccc(Cl)cc3s2)c(OC)c1 |
| CHEMBL1979577 | Cc1cccc(NC(=O)Nc2ccc(cc2)c3csc4c(cnc(N)c34)C#CC(C)(C)N)c1 |
| CHEMBL207253 | CCCON1c2ccc(Cl)cc2C(=O)c3c(C)nn(C)c13 |
| CHEMBL1992937 | Cc1ccc(Nc2nc3ccc(cc3[nH]2)[N+](=O)[O-])nc1 |
| CHEMBL1975208 | CC(=O)NC1CCN(C1)c2nc3NC=C(C(=O)O)C(=O)c3c(C)c2F |
| CHEMBL253969 | NC(=O)c1c(NC(=O)NCCCCN2CCCC2)snc1OCc3c(F)cc(Br)cc3F |
| CHEMBL1992342 | CCCS(=O)(=O)Nc1ccc(cc1)c2ccc3[nH]nc(N)c3c2 |
| CHEMBL1970314 | NC(=O)c1cc(cc2cccnc12)c3cccc(N)c3 |
| CHEMBL1972659 | Cc1c(O)ccc2C(=NNC(=O)Cc3cccc4ccccc34)CCCc12 |
| CHEMBL2004934 | COc1cccc(c1)C(C)NC(=O)c2ccc(c(F)c2)c3ccncc3 |
| CHEMBL1990288 | CC(Nc1nccc(n1)c2c(nc3occn23)c4ccc(F)cc4)c5ccccc5 |
| CHEMBL573339 | Oc1cccc(c1)c2nc(N3CCOCC3)c4oc5ncccc5c4n2 |
| CHEMBL1996817 | CCCCOc1ncnc2[nH]cc(c3ccccc3)c12 |
| CHEMBL1988717 | Cn1cc(cn1)c2cnn3c(N)c(cnc23)c4ccc(NC(=O)Nc5cccc(c5)C(F)(F)F)cc4 |
| CHEMBL2006263 | Cc1nc2ccc(NS(=O)(=O)c3ccc(N)cc3)cc2nc1C |
| CHEMBL2003341 | OCC(NC(=O)c1ccc(cc1)c2ccncc2)c3cc(Cl)cc(Cl)c3 |
| CHEMBL1991395 | CC(C)(CO)CNc1nccc(n1)c2c(nc3cnccn23)c4ccc(F)cc4 |
| CHEMBL1996931 | OCC(Cc1ccc(Cl)cc1)NC(=O)c2ccc(cc2)c3ccncc3 |
| CHEMBL1973348 | NC(COc1cncc(c1)c2ccc(N)c(c2)C(=O)c3cccc(Cl)c3)Cc4c[nH]c5ccccc45 |
| CHEMBL95692 | COc1nccc(n1)c2c(ncn2C3CCNCC3)c4ccc(F)cc4 |
| CHEMBL1994040 | CC(C)(O)CNc1nccc(n1)c2c(nc3c(CC4CC4)nccn23)c5ccc(F)cc5F |
| CHEMBL1994074 | Brc1ccc2[nH]nc(c3ccccc3)c2c1 |
| CHEMBL2004892 | CS(=O)(=O)Nc1ccc(cc1)c2ccc3[nH]nc(N)c3c2 |
| CHEMBL178737 | O=C(N1CCOCC1)N2CCn3cc(C4=C(C(=O)NC4=O)c5cnc6ccccn56)c7cccc(C2)c37 |
| CHEMBL1082440 | CC1CNCCCN1S(=O)(=O)c2cccc3cncc(C)c23 |
| CHEMBL2007372 | CNc1nccn2c(c3ccnc(NCC(C)(C)CO)n3)c(nc12)c4ccc(F)cc4 |
| CHEMBL1982874 | Nc1nc2ccc(cc2n1CC3CC3)c4c(nc5sccn45)c6ccc(F)cc6 |
| CHEMBL2007044 | CCN(CC)CC#Cc1ccc2Cc3c(n[nH]c3c2c1)c4ccc(nc4)C#N |
| CHEMBL1997025 | NC(=O)c1cccc2[nH]c(nc12)c3ccc(cc3F)C4CCCNC4 |
| CHEMBL116070 | NC(=O)c1cc2c(Oc3ccc(Br)cc3)cncc2s1 |
| CHEMBL1090356 | O=C(Cc1ccccc1)Nc2cccc(c2)c3nc4sccn4c3c5ccnc(Nc6ccc(cc6)N7CCOCC7)n5 |
| CHEMBL424872 | CCOc1nc(cc(N)c1C#N)C(=O)NCc2ccc(cc2)S(=O)(=O)C |
| CHEMBL1988153 | Cc1oc(nn1)c2cc3c(Oc4ccc(cc4)C(F)(F)F)cncc3s2 |
| CHEMBL1983923 | CN1CCN(CC1)c2cc(Nc3cc(C)[nH]n3)nc(C=Cc4ccccc4)n2 |
| CHEMBL1986781 | CC(=O)N1CCC(CC1)Nc2nccc(n2)c3c(nc4occn34)c5ccc(F)cc5 |
| CHEMBL474432 | COc1cc2ncn(c3cc(OCc4ccccc4C(F)(F)F)c(s3)C(=O)O)c2cc1OC |
| CHEMBL1996979 | Fc1cccc(F)c1C(=O)Nc2cnc3[nH]cc(c4ccccc4)c3c2 |
| CHEMBL1972125 | NC(COc1cncc(c1)c2ccc(N)c(c2)C(=O)c3ccccc3)Cc4c[nH]c5ccccc45 |
| CHEMBL1614705 | OCC(=O)N1CCC(CC1)c2[nH]nc(c3ccc(Cl)cc3F)c2c4ccncn4 |
| CHEMBL2004647 | CNC1CCN(C1)c2nc3NC=C(C(=O)O)C(=O)c3c(C)c2Br |
| CHEMBL2000508 | Fc1ccc(cc1)c2nc3sccn3c2c4ccncc4 |
| CHEMBL2004692 | Nc1nonc1n2nnc(C(=O)NN=Cc3cccs3)c2c4ccccc4 |
| CHEMBL1988594 | Nc1ncnc2scc(C(=O)Nc3ccc(NC(=O)Nc4ccccc4)cc3)c12 |
| CHEMBL1981410 | CNc1ncnc2NCCC(=Nc12)c3ccc(NC(=O)Nc4cccc(c4)C(F)(F)F)cc3 |
| CHEMBL1964444 | Cc1ccc2nccc(NC(=O)Nc3cccc(n3)C(F)(F)F)c2c1 |
| CHEMBL1991725 | CN(C)CC(=O)NC(COc1cncc(c1)c2ccc3cnccc3c2)Cc4c[nH]c5ccccc45 |
| CHEMBL1973860 | OCCNc1cc2cc(ccc2cn1)c3ccsc3 |
| CHEMBL387971 | COCCOCC#Cc1cc(cs1)c2n[nH]c3c4ccc(CN5CCN(C)CC5)cc4C(=O)c23 |
| CHEMBL1975138 | OC(C(=O)NNC(=S)Nc1ccc(F)cc1)(c2ccccc2)c3ccccc3 |
| CHEMBL2002240 | CC(=O)Nc1cc(N)c(C#N)c(n1)c2ccc3ccccc3c2 |
| CHEMBL244378 | OC(=O)c1ccccc1Nc2ccnc(Nc3ccc4cn[nH]c4c3)n2 |
| CHEMBL1968127 | CN(C)CC(C)(C)CNc1nccc(n1)c2c(nc3occn23)c4ccc(F)cc4 |
| CHEMBL1989957 | OCCNc1cc2cc(ccc2cn1)c3ccccc3 |
| CHEMBL3736278 | FC(F)(F)c1cccc(c1)C(=O)Nc2nc3cc(CN4CCCCC4)ccc3n2c5ccccc5 |
| CHEMBL260933 | CCCN1C(=O)N(CC)c2nc([nH]c2C1=O)c3cnn(Cc4cccc(c4)C(F)(F)F)c3 |
| CHEMBL1991429 | O=C(Cc1ccccc1)Nc2cccc(c2)c3nc4sccn4c3c5ccnc(Nc6cccc(c6)N7CCCC7=O)n5 |
| CHEMBL1974664 | COc1ccc(C=NNc2ncnc3c4ccccc4[nH]c23)cc1OC |
| CHEMBL1983025 | CCC(N)(CC)C#Cc1cnc(N)c2c(csc12)c3ccc(NC(=O)Nc4cccc(C)c4)cc3 |
| CHEMBL1978099 | CNC(=O)COc1ccc(Nc2nc(Nc3ccc(C)c(c3)S(=O)(=O)N)ncc2F)cc1 |
| CHEMBL1982466 | Cc1ccc(NC(=O)Nc2ccc(cc2)c3csc4c(cnc(N)c34)c5cnn(C)c5)cc1 |
| CHEMBL3734854 | OC[C@@H]1CC[C@@H](CC1)n2c(NC(=O)c3cccc(c3)[N+](=O)[O-])nc4cc(CN5CCCCC5)ccc24 |
| CHEMBL1982610 | Clc1cccc(c1)c2cn3C(=O)NC=C(I)c3n2 |
| CHEMBL1979516 | CCNC(=O)c1cnc(N)c2c(csc12)c3ccc(NC(=O)Nc4cccc(F)c4)cc3 |
| CHEMBL1989518 | NCCOc1cncc(C=Cc2ccncc2)c1 |
| CHEMBL1991678 | Cc1nccn2c(c3ccnc(NCC(C)(C)CO)n3)c(nc12)c4ccc(F)cc4F |
| CHEMBL1969755 | OCC(Cc1ccccc1)NC(=O)c2ccc(cc2F)c3ccncc3 |
| CHEMBL392642 | CSc1ccccc1CNc2ncc(c(NCC3CCC(CN)CC3)n2)[N+](=O)[O-] |
| CHEMBL1987982 | FC(F)(F)Oc1ccc(cc1)c2ccc3[nH]cc(C4=CCNCC4)c3c2 |
| CHEMBL1998551 | CC(C)(O)c1nnc2ccc(nn12)c3c(nc4CCCn34)c5ccc(F)cc5F |
| CHEMBL1190711 | COc1ccc(cc1OC)c2cc3nccn3c(Nc4ncccc4C(=O)N)n2 |
| CHEMBL1974870 | Cc1n[nH]c2ccc(cc12)c3cncc(OCC(N)Cc4cccc(Cl)c4)c3 |
| CHEMBL1242373 | Cc1cccc(NC(=O)Nc2ccc3c(CCc4sc5ncnc(N)c5c34)c2)c1 |
| CHEMBL1990482 | NC(=O)c1cccc2[nH]c(nc12)c3ccncc3 |
| CHEMBL565612 | CN1CCN(CC1)c2nc(C3=C(C(=O)NC3=O)c4c[nH]c5ccccc45)c6ccccc6n2 |
| CHEMBL2004615 | CSc1nn(c(N)c1C(=O)N)c2ccccn2 |
| CHEMBL583042 | Cc1cnc(Nc2ccc(F)cc2Cl)nc1c3c[nH]c(c3)C(=O)N[C@H](CO)c4cccc(Cl)c4 |
| CHEMBL1988537 | COc1ccc(cc1)C(=O)N2c3ccccc3Sc4c(Cl)cncc24 |
| CHEMBL2007592 | O=C1Nc2sc3CCCCc3c2c4nc(nn14)c5ccccc5 |
| CHEMBL2001257 | CCN(CC)CC#Cc1cnc(N)c2c(csc12)c3ccc(NC(=O)Nc4cccc(C)c4)cc3 |
| CHEMBL1973142 | CC(=O)Nc1c(sc2ccc(Cl)c(Cl)c12)C(=O)N |
| CHEMBL1967560 | CCN1N(C2CCCC2)c3nc(Nc4ccc(cc4OC)C(=O)NC5CCN(C)CC5)ncc3N(C)C1=O |
| CHEMBL2006156 | CC(C)N(C(C)C)C(=O)Nc1ccc2nc(c3occc3)c(nc2c1)c4occc4 |
| CHEMBL2001751 | NC(COc1cncc(c1)c2ccc3NC(=O)C(=Cc4occc4)c3c2)Cc5c[nH]c6ccccc56 |
| CHEMBL1985406 | CCCC(=O)Nc1n[nH]c2ccc(cc12)c3cccc(F)c3F |
| CHEMBL1986186 | CC(NC1=C(Nc2ccncc2)C(=O)C1=O)C(C)(C)C |
| CHEMBL1976732 | CC(C)(O)Cc1nnc2ccc(nn12)c3c(nc4occn34)c5ccc(F)cc5F |
| CHEMBL2000114 | CSc1c[nH]c2ncnc(NCCCO)c12 |
| CHEMBL3735673 | O[C@@H]1CC[C@H](CC1)n2c(NC(=O)c3cccc(c3)[N+](=O)[O-])nc4cc(CN5CCCCC5)ccc24 |
| CHEMBL2003817 | FC(F)(F)c1ccc2c(NC(=O)Nc3cnccn3)ccnc2c1 |
| CHEMBL1982465 | Cc1cccc(NC(=O)Nc2ccc(cc2)c3csc4c(cnc(N)c34)C#CCN5CCOCC5)c1 |
| CHEMBL1994321 | Cc1cccc(NC(=O)Nc2ccc(cc2)c3csc4c(cnc(N)c34)C#CCNS(=O)(=O)C)c1 |
| CHEMBL1982957 | NC(=O)c1cccc(c1)c2cnc3[nH]cc(c4ccccc4)c3c2 |
| CHEMBL2006836 | CC1=NN(C(=O)C1=Cc2c(C)c(C#N)c3nc4ccccc4n3c2O)c5ccc(cc5)C(=O)O |
| CHEMBL1967544 | CN(C)c1cccc2c(cccc12)S(=O)(=O)N(CCN)c3cncc(c3)c4ccc5cnccc5c4 |
| CHEMBL1973098 | COc1cc(C=C2SC(=S)NC2=O)ccc1O |
| CHEMBL1991063 | Cc1n[nH]c2ccc(cc12)c3cncc(OCC(N)Cc4ccc(Cl)c(Cl)c4)c3 |
| CHEMBL1983111 | CC(=O)NCC(=O)N1C2CCC1c3cc(Nc4ncc(c(NC5CCC5)n4)C(F)(F)F)ccc23 |
| CHEMBL491473 | COc1cc2c(Oc3ccc4[nH]c(C)cc4c3F)ncnc2cc1OCCCN5CCCC5 |
| CHEMBL210963 | CCOc1nc(NC(=O)Cc2cc(OC)c(cc2OC)S(=O)(=O)C)cc(N)c1C#N |
| CHEMBL223367 | OCCCc1cc2OCCCCCOc3nc(NC(=O)Nc2cc1Cl)cnc3C#N |
| CHEMBL1971021 | Cc1cc(C)cc(NC(=O)Nc2ccc(c3cccc4C(=O)NCc34)c(c2)C(F)(F)F)c1 |
| CHEMBL1965988 | Cc1cccc(NC(=O)Nc2ccc(cc2)c3csc4c(cnc(N)c34)C#CCN)c1 |
| CHEMBL1969523 | CC(NC1=C(Nc2ccnc(Nc3cccc(C)c3)n2)C(=O)C1=O)C(C)(C)C |
| CHEMBL1997839 | CCOC(=O)C1=CN(c2ccc(O)cc2C)c3cc(ccc3C1=O)c4ccccc4 |
| CHEMBL1989646 | Cc1c(nnn1Cc2ccccc2)c3ccc4[nH]nc(N)c4c3 |
| CHEMBL1992740 | CCN1CCN(CCCC(=O)Nc2n[nH]c3nnc(cc23)c4cccc(F)c4F)CC1 |
| CHEMBL1984162 | COCCOC1CCC(CC1)n2nc(c3ccc(Nc4oc5c(C)cc(C)cc5n4)cc3)c6c(N)ncnc26 |
| CHEMBL1972119 | FC(F)(F)c1cccc2c(NC(=O)Nc3cccc(n3)N4CCOCC4)ccnc12 |
| CHEMBL50894 | [O-][N+](=O)c1ccc2[nH]c3c(CC(=O)Nc4ccccc34)c2c1 |
| CHEMBL1970913 | CCCC(=O)Nc1nn(C(=O)CC)c2nc3ccccc3cc12 |
| CHEMBL1990821 | Cc1cc(C)c(C)c(OCCCc2c([nH]c3c(cccc23)c4ccccc4C)C(=O)O)c1 |
| CHEMBL234085 | CC1CCN(CC1N(C)c2ncnc3[nH]ccc23)C(=O)CC#N |
| CHEMBL1969735 | CC(C)(CO)CNc1nccc(n1)c2c(nc3c(Cl)nccn23)c4ccc(F)cc4 |
| CHEMBL1090360 | O=C(Cc1ccccc1)Nc2cccc(c2)c3nc4sccn4c3c5ccnc(Nc6cccc(c6)N7CCOCC7)n5 |
| CHEMBL1682546 | C(Nc1nc(cs1)c2ccc3[nH]ncc3c2)c4ccccc4 |
| CHEMBL226403 | O=C1NCCc2[nH]c(cc12)c3ccnc(c3)c4cnc5ccccc5c4 |
| CHEMBL1988300 | Nc1ccc(cc1)c2csc3c(C=Cc4nc5ccccc5[nH]4)cnc(N)c23 |
| CHEMBL1964413 | FC(F)(F)c1ccc(Cn2cc(nn2)c3ccc4[nH]ncc4c3)c(c1)C(F)(F)F |
| CHEMBL1992242 | Cc1cc(C)cc(NC(=O)Nc2ccc(cc2)c3cccc4sncc34)c1 |
| CHEMBL2000393 | Fc1ccc2ncn(c3ncc4NC(=O)N(C5CCOc6c(F)cccc56)c4n3)c2c1 |
| CHEMBL1993941 | CCCn1cc(cn1)c2cnc(N)c3c(csc23)c4ccc(NC(=O)Nc5cccc(F)c5)cc4 |
| CHEMBL1986499 | Nc1n[nH]c2ccc(cc12)c3nnn(Cc4ccccc4)c3c5ccc(F)cc5 |
| CHEMBL468280 | CCN1C2=C(C(=O)ON2)C(=O)c3cc(F)c(Cl)cc13 |
| CHEMBL1972276 | CCN(CC)CCCNc1cccc(n1)c2nc3c(cccc3[nH]2)C(=O)N |
| CHEMBL1986263 | CCN1C(=O)SC(=C1C)c2ccnc(Nc3cccc(OC)c3)n2 |
| CHEMBL1970104 | CC(C)(C)c1ccc2nc([nH]c2c1)c3n[nH]c4ccccc34 |
| CHEMBL1290072 | COc1ccc(CNc2ccnc3oc4ccccc4c23)cc1 |
| CHEMBL377383 | CCOc1nc(cc(N)c1Cl)C(=O)NCc2ccc(cc2)S(=O)(=O)C |
| CHEMBL560813 | OC(=O)c1ccc2cc(NC(=O)Nc3cnc(cn3)C#N)ccc2c1 |
| CHEMBL1230122 | CN1C(=O)C(=Cc2cnc(NC3CCOCC3)nc12)Oc4ccc(F)cc4F |
| CHEMBL1992634 | Brc1cnc2[nH]cc(c3ccccc3)c2c1 |
| CHEMBL1979252 | FC(F)(F)c1cccc(CNC2=C(Nc3ccncc3)C(=O)C2=O)c1 |
| CHEMBL1982122 | OCCC(Nc1nc2ccc(cc2s1)c3ccncc3)c4ccccc4 |
| CHEMBL1997872 | O=C(N1CCOCC1)c2cccc(c2)c3cnc4[nH]ccc4c3 |
| CHEMBL2004311 | Cc1cc(C)cc(NC(=O)Nc2ccc(c(C)c2)c3cccc4C(=O)NCc34)c1 |
| CHEMBL1965131 | Cc1ccc(cc1)C(=O)Nc2c(sc3ccc(Cl)c(Cl)c23)C(=O)N |
| CHEMBL1974702 | CC1=Nc2c(sc3c(Br)ccc(Cl)c23)C(=O)O1 |
| CHEMBL210887 | OCCCn1c(NC(=O)c2cccc(c2)[N+](=O)[O-])nc3ccccc13 |
| CHEMBL1992536 | COc1ccc(CNC2=C(Nc3ccc4[nH]ncc4c3)C(=O)C2=O)cc1 |
| CHEMBL1990912 | OCCNc1cc2cc(ccc2cn1)c3cccnc3 |
| CHEMBL1988995 | CC(C)c1nnc2ccc(nn12)c3c(nc4occn34)c5ccc(F)cc5F |
| CHEMBL458997 | CNC(=O)c1ccccc1Nc2nc(Nc3ccc(cc3OC)N4CCOCC4)ncc2Cl |
| CHEMBL1986139 | Clc1csc2ncnc(Nc3ccccc3)c12 |
| CHEMBL2000568 | CC(=O)c1cccc(c1)c2cnc3[nH]ccc3c2 |
| CHEMBL1993722 | Nc1n[nH]c2cccc(c3ccc(NC(=O)Nc4cc(ccc4F)C(=O)O)cc3)c12 |
| CHEMBL1995927 | CCNc1nc2cc(Cl)c(OC)cc2nc1NCC |

**Supplementary Table S2.** Inhibition rates and docking scores of 38 compounds selected from traditional VS results.

| **Compound** | **Glide SP** | **Glide XP** | **Inhibition/%** |
| --- | --- | --- | --- |
| D365-0053 | -9.92 | -10.58 | -14.7 |
| G856-0738 | -11.88 | -11.90 | -0.7 |
| G807-0668 | -10.38 | -11.76 | -4.2 |
| Y020-2652 | -10.55 | -11.59 | -3.6 |
| D451-1432 | -10.25 | -11.08 | 3.2 |
| D715-1652 | -10.13 | -11.57 | -1.3 |
| 8019-0047 | -9.98 | -11.39 | -13.9 |
| Y041-8246 | -10.45 | -11.14 | 85.5 |
| M008-0146 | -9.17 | -10.60 | -0.7 |
| G528-0626 | -9.06 | -10.58 | -2.2 |
| S664-4928 | -9.54 | -10.33 | -12.6 |
| F779-0762 | -9.75 | -9.85 | 18.0 |
| F926-1699 | -9.40 | -9.56 | 1.0 |
| G856-8131 | -9.31 | -9.06 | -15.6 |
| F410-1220 | -10.31 | -10.85 | 13.0 |
| D451-5164 | -10.64 | -9.18 | -3.8 |
| E594-0144 | -9.18 | -9.27 | -3.2 |
| E822-0619 | -9.30 | -9.90 | 5.4 |
| E882-1152 | -8.95 | -9.06 | 2.0 |
| F248-0097 | -9.98 | -10.30 | -2.2 |
| F265-0010 | -9.60 | -9.93 | 1.5 |
| F416-0072 | -10.24 | -10.80 | -7.2 |
| F808-0564 | -9.30 | -10.13 | -6.3 |
| G298-0210 | -9.43 | -9.09 | -3.2 |
| G856-8135 | -9.90 | -9.12 | -2.4 |
| G856-8153 | -9.64 | -9.41 | -8.1 |
| K231-1211 | -9.06 | -9.10 | -10.5 |
| K832-1694 | -9.83 | -10.16 | -7.7 |
| L309-0452 | -9.08 | -9.15 | -1.9 |
| S562-0067 | -9.90 | -11.00 | 2.6 |
| S576-0135 | -10.03 | -9.83 | -14.7 |
| T408-2201 | -9.75 | -9.25 | 4.9 |
| V018-9455 | -9.29 | -9.67 | 4.3 |
| V030-8810 | -9.52 | -9.21 | -13.3 |
| Y041-1079 | -9.48 | -10.46 | 8.5 |
| Y041-4214 | -9.13 | -10.30 | -0.2 |
| Y041-4243 | -9.49 | -9.89 | -12.6 |
| Y041-7737 | -9.37 | -9.43 | 3.4 |

**Supplementary Table S3.** Active dataset and inactive dataset for model construction.

| ChEMBL ID | Smiles |
| --- | --- |
| Actives: |  |
| CHEMBL1973348 | c1ccc(Cl)cc1C(=O)c2cc(ccc2N)-c3cc(cnc3)OCC(N)Cc4c[nH]c(c45)cccc5 |
| CHEMBL1971694 | CCCCNC(=N1)C(=NS1=O)Nc2ccc(F)cc2 |
| CHEMBL1972152 | C1SCc(c2=O)c1[nH]c(n23)cc(n3)-c4occc4 |
| CHEMBL1975357 | c1cccc(c12)n(C(=O)OC(C)(C)C)cc2-c(cnc3)n3C4CCCCC4 |
| CHEMBL1998551 | c1cc(F)cc(F)c1-c(nc(n23)CCC3)c2-c(n4)ccc(n45)nnc5C(O)(C)C |
| CHEMBL3735504 | FC(F)(F)c(c1)cccc1C(=O)Nc(n2[C@@H]3CC[C@@H](CC3)CO)nc(c24)cc(cc4)NC(=O)C(C)(C)C |
| CHEMBL3734814 | C1C[C@@H](CO)CC[C@H]1n2c(nc(c23)ccc(c3)CO)NC(=O)c4cccc(c4)C(F)(F)F |
| CHEMBL2000481 | NCCNS(=O)(=O)c1cc(ccc1)C(=O)Nc(cc2)ccc2-c3n[nH]c(=O)c(c34)cccc4 |
| CHEMBL2004311 | C1NC(=O)c(c12)cccc2-c3c(C)cc(cc3)NC(=O)Nc4cc(C)cc(c4)C |
| CHEMBL1290072 | c1cc(OC)ccc1CNc2ccnc(c23)oc4c3cccc4 |
| CHEMBL1977713 | c1cc(Cl)ccc1CNC(=O)Nc2cccc(c23)[nH]nc3 |
| CHEMBL178737 | C1COCCN1C(=O)N(C2)CCn3cc(c(c3c24)ccc4)C5=C(C(=O)NC5=O)c6cnc(n67)cccc7 |
| CHEMBL2002682 | NCCOCCOCCNC(=O)c([nH]1)c(c(c12)cccc2-c3c(Cl)cccc3C)CCCOc4cccc(c45)cccc5 |
| CHEMBL1988163 | c1ccc(S(=O)(=O)N)cc1Nc(n2)ncc(c23)ccn3-c4ccccc4 |
| CHEMBL1375418 | c1ccccc1-c2nnc(n23)ccc(n3)NCc4ccc(C)cc4 |
| CHEMBL526133 | c1n[nH]c(c12)ccc(c2)-c3cc([nH]c(n3)=O)-c4ccccc4 |
| CHEMBL1965845 | c1ccc(OC)cc1C(C)NC(=O)N2CC=C(CC2)c3c[nH]c(c34)nccc4 |
| CHEMBL2001957 | FC(F)(F)COc(ncc1)c(n12)nc(-c3ccc(F)cc3)c2-c4nc(ncc4)NCC(C)(C)CO |
| CHEMBL1995813 | c1ccnc(c12)[nH]cc2-c3nc(ncc3)NC4C(N)CCCC4 |
| CHEMBL1996923 | Clc(c1)c(Cl)cc(c12)[nH]nn2 |
| CHEMBL535331 | c1cccc(c12)nc([nH]2)N/N=C/c3c[nH]c(c34)cccc4 |
| CHEMBL1972849 | c1cc(F)cc(F)c1-c(nc(n23)occ3)c2-c(n4)ccc(n45)nnc5C(C)(C)CO |
| CHEMBL1999718 | FC(F)(F)c(cc1)cc(c12)nccc2NC(=O)Nc3ncc(C#N)cc3 |
| CHEMBL1991143 | Clc(c1)ccc(n2O)c1c(=O)c(c23)c(C)nn3-c4ccc(C)cc4 |
| CHEMBL1995927 | COc(c1)c(Cl)cc(c12)nc(NCC)c(n2)NCC |
| CHEMBL1990635 | FC(F)(F)Oc(cc1)ccc1-c(c2)ccc(c23)[nH]c(c3)CCN4C(C)CCC4 |
| CHEMBL1984788 | O=C(N)c1cc(Cl)cc(c12)[nH]c(n2)-c3c(F)cc(cc3)C4CNCCC4 |
| CHEMBL1972258 | NCCCOc(cnc1)cc1/C=C/c2ccncc2 |
| CHEMBL2001668 | O=c1[nH]cc(I)c(n12)nc(c2)-c3ccccc3 |
| CHEMBL1999126 | c1cccc2c1nc(n23)c(C#N)c(C)c(c3O)\C=C(\C4=O)C(C)=NN4c5ccccc5 |
| CHEMBL2005112 | c1ccc(Br)cc1N2N=C(C)C(\C2=O)=C/c(c3O)c(C)c(C#N)c(n34)nc5c4cccc5 |
| CHEMBL1966143 | c1cccc2c1nc(n23)cc(C)c(c3O)\C=C(\C4=O)C(C)=NN4c5ccc(cc5)C(=O)O |
| CHEMBL1964777 | O=C(N)c(c1)sc(c12)cncc2Oc3cc(F)ccc3 |
| CHEMBL1965988 | Nc1ncc(C#CCN)c(c12)scc2-c3ccc(cc3)NC(=O)Nc4cc(C)ccc4 |
| CHEMBL1984633 | c1cnccc1-c(nc2)sc2C(=O)NC(C)c3cc(OC)ccc3 |
| CHEMBL1987533 | c1cccc(Cl)c1CC(=O)Nc(c2)ccc(c23)cn[nH]3 |
| CHEMBL1991356 | c1cccc2c1[nH]c(=O)c(n23)cc(c3)-c4cc(OC)ccc4 |
| CHEMBL2007372 | c1cnc(NC)c(n12)nc(-c3ccc(F)cc3)c2-c4nc(ncc4)NCC(C)(C)CO |
| CHEMBL1974157 | c1cc(O)c(O)cc1/C=C(C#N)\C(=O)NCc2ccccc2 |
| CHEMBL1979516 | Nc1ncc(C(=O)NCC)c(c12)scc2-c3ccc(cc3)NC(=O)Nc4cc(F)ccc4 |
| CHEMBL1977604 | CC1CSc2c(C(=O)O)c(=O)c(c3n12)cc(F)c(c3F)N4CC(N)CC4 |
| CHEMBL1966816 | FC(F)(F)c1nc(ccc1)NC(=O)Nc2ccnc(c23)c(F)ccc3 |
| CHEMBL1974254 | Cn1ncc(c1)-c(cnc2N)c(c23)scc3-c4ccc(cc4)NC(=O)Nc5ccc(cc5)OCC |
| CHEMBL1970142 | CNC(=O)C(C)n1ncc(c1)-c(cnc2N)c(c23)scc3-c4ccc(cc4)NC(=O)Nc5ccc(C)cc5 |
| CHEMBL1972820 | c1cccc(c12)C(=O)N(C2=O)c3cc(O)c(cc3)C(=O)O |
| CHEMBL3736036 | FC(F)(F)c(c1)cccc1C(=O)Nc(n2[C@@H]3CC[C@@H](CC3)CO)nc(c24)cc(cc4)CN5CCCCC5 |
| CHEMBL3736465 | FC(F)(F)c(c1)cccc1C(=O)Nc(n2-c3ccc(cc3)CCO)nc(c24)cc(cc4)CN5CCCCC5 |
| CHEMBL3736278 | FC(F)(F)c(c1)cccc1C(=O)Nc(n2-c3ccccc3)nc(c24)cc(cc4)CN5CCCCC5 |
| CHEMBL1972568 | c1ncc(OC)cc1/C=C/c2ccncc2 |
| CHEMBL260933 | CCCn(c1=O)c(=O)n(CC)c(c12)nc([nH]2)-c3cnn(c3)Cc4cccc(c4)C(F)(F)F |
| CHEMBL2002373 | c1cnccc1-c(n2CCCO)c(nc2)-c3ccccc3 |
| CHEMBL1984044 | O=C(N)c1cccc(c12)[nH]c(n2)-c3ccc(cc3)OCCN(CC)CC |
| CHEMBL1973540 | s1cnc(c1c23)ccc3NC(=O)C\2=C/Nc(cc4)ccc4S(=O)(=O)Nc5ncccc5 |
| CHEMBL1727312 | c1cccc(NC2=O)c1Nc(c23)cccc3 |
| CHEMBL244378 | c1n[nH]c(c12)cc(cc2)Nc3nccc(n3)Nc4c(C(=O)O)cccc4 |
| CHEMBL207253 | c1c(Cl)ccc(c12)n(OCCC)c3c(c2=O)c(C)nn3C |
| CHEMBL1984274 | c1ccccc1N2N=C(c3ccccc3)/C(C2=O)=N\Nc4cccc(c45)c(=O)[nH][nH]c5=O |
| CHEMBL1979176 | FC(F)(F)c1cc(ncc1)NC(=O)Nc2ccnc(c23)cc(OC)cc3 |
| CHEMBL1971021 | C1NC(=O)c(c12)cccc2-c3c(C(F)(F)F)cc(cc3)NC(=O)Nc4cc(C)cc(c4)C |
| CHEMBL1998159 | FC(F)(F)c1cc(ccc1)CC(N)COc(cnc2)cc2-c(c3)ncc(c34)[nH]nc4C |
| CHEMBL539474 | c1ccc(n12)c(=O)[nH]c3c2ccc(c3)CN4CCCC4=O |
| CHEMBL1977148 | OCCn1ncc(c1)-c(cnc2N)c(c23)scc3-c4ccc(cc4)NC(=O)Nc5ccc(OC)cc5 |
| CHEMBL2006631 | c1cccc(S(=O)(=O)N(C)C)c1-c(c2)ccc(c23)C(=O)Nc4c(N3)cccc4 |
| CHEMBL1989805 | c1ccoc1\C=c(c2=O)\sc(n23)=C(C(=O)N)C(C(C#N)=C3N)c4occc4 |
| CHEMBL1999496 | c1cnccc1NC(=O)C(C2)C2(c3cc(C)ccc3)c4cc(C)ccc4 |
| CHEMBL1974803 | Clc1cc(Cl)cc(c12)[nH]c(=O)c(O)c2-c3c(O)ccc(Cl)c3 |
| CHEMBL1999428 | c1ccccc1C(C)NC(=O)c2ccc(cc2)-c3ccncc3 |
| CHEMBL1991434 | c1cc(Br)ccc1C(=NOC2=O)/C2=C/c3cc(OC)c(OC)cc3 |
| CHEMBL1996587 | c1cc(F)cc(F)c1-c(nc(n23)CCC3)c2-c(n4)ccc(n45)nnc5C(C)(C)CO |
| CHEMBL3735719 | O=[N+]([O-])c(c1)cccc1C(=O)Nc(n2-c3ccccc3)nc(c24)cc(cc4)CN5CCCCC5 |
| CHEMBL3734854 | O=[N+]([O-])c(c1)cccc1C(=O)Nc(n2[C@@H]3CC[C@@H](CC3)CO)nc(c24)cc(cc4)CN5CCCCC5 |
| CHEMBL3735673 | O=[N+]([O-])c(c1)cccc1C(=O)Nc(n2[C@H]3CC[C@H](O)CC3)nc(c24)cc(cc4)CN5CCCCC5 |
| CHEMBL2003341 | Clc(c1)cc(Cl)cc1C(CO)NC(=O)c2ccc(cc2)-c3ccncc3 |
| CHEMBL1969755 | c1cnccc1-c(cc2)cc(F)c2C(=O)NC(CO)Cc3ccccc3 |
| CHEMBL1969735 | c1cnc(Cl)c(n12)nc(-c3ccc(F)cc3)c2-c4nc(ncc4)NCC(C)(C)CO |
| CHEMBL1971029 | c1cccc(c1S(=O)(=O)NC)Nc2nc(ncc2Br)Nc3c(OC)ccc(c3)N4CCN(CC4)C(=O)C |
| CHEMBL1988838 | O=S(=O)(N)c1cc(ccc1)Nc(ncc2C)nc2Nc3ccc(cc3)OCC(=O)N |
| CHEMBL1978099 | O=S(=O)(N)c1c(C)ccc(c1)Nc2nc(c(F)cn2)Nc3ccc(cc3)OCC(=O)NC |
| CHEMBL1977374 | c1cc(F)cc(F)c1-c(nc(n23)CCC3)c2-c(n4)ccc(n45)nnc5C(C)C |
| CHEMBL1989474 | c1cc(F)ccc1-c(nn2C)c(c2)-c3ncncc3 |
| CHEMBL1988995 | c1cc(F)cc(F)c1-c(nc(n23)occ3)c2-c(n4)ccc(n45)nnc5C(C)C |
| CHEMBL1982980 | c1ccc(Cl)c(Cl)c1OCCCc(c([nH]2)C(=O)O)c(c23)cccc3C(C)=C(C)C |
| CHEMBL1987982 | FC(F)(F)Oc(cc1)ccc1-c(c2)ccc(c23)[nH]cc3C4=CCNCC4 |
| CHEMBL1997534 | Cc1n[nH]c(c12)ccc(c2)-c3sc(cc3C)C(=O)NC(C)c4cc(OC)ccc4 |
| CHEMBL2004513 | c1cccc(N2)c1NC(=O)c(c23)ccc(c3)-c4c(C(=O)OC)cccc4 |
| CHEMBL1972119 | FC(F)(F)c1cccc(c12)c(ccn2)NC(=O)Nc(ccc3)nc3N4CCOCC4 |
| CHEMBL1997872 | C1COCCN1C(=O)c(ccc2)cc2-c(c3)cnc(c34)[nH]cc4 |
| CHEMBL1964290 | c1cnc(N)nc1-c(cc2C(=O)N)[nH]c2-c3c(C)c(C)ccc3 |
| CHEMBL2004647 | C1CC(NC)CN1c(c(Br)c2C)nc(c23)[nH]cc(c3=O)C(=O)O |
| CHEMBL1972183 | C1CCC(N1C)COc2cc(cnc2)CCc3ccccc3 |
| CHEMBL1972276 | O=C(N)c1cccc(c12)[nH]c(n2)-c3nc(ccc3)NCCCN(CC)CC |
| CHEMBL2004365 | c1ccsc1\C=c(c2=O)\sc(n23)=C(C(=O)N)C(C(=C3N)C(=O)OCC)c4sccc4 |
| CHEMBL1991800 | C1CC1Cc(ncc2)c(n23)nc(-c4ccc(F)cc4)c3-c5nc(ncc5)NCC(C)(C)CO |
| CHEMBL1989646 | [nH]1nc(N)c(c12)cc(cc2)-c(c3C)nnn3Cc4ccccc4 |
| CHEMBL1991429 | c1ccccc1CC(=O)Nc(ccc2)cc2-c(nc(n34)scc4)c3-c5nc(ncc5)Nc6cc(ccc6)N7C(=O)CCC7 |
| CHEMBL462120 | c1cc(Cl)cc(c12)c(c(N)c(=O)[nH]2)-c3ccccc3 |
| CHEMBL1997503 | CC(C)Oc(cc1)c(Cl)cc1-c2nc(on2)-c3ccc(cc3)NC4CC(CC4)C(=O)O |
| CHEMBL1992740 | c1ccc(F)c(F)c1-c(c2)nnc(c23)[nH]nc3NC(=O)CCCN4CCN(CC4)CC |
| CHEMBL2001646 | c1cccc(C)c1-c2cccc(c23)n(c(C(=O)O)c3Nc4ccccc4)CCCOc5cccc(c56)cccc6 |
| CHEMBL1987793 | c1cccc(C)c1-c2c(C(=O)O)n(c(c23)cccc3)CCCOc4cccc(c45)cccc5 |
| CHEMBL1970735 | CCCOc(c1C#N)nc(NC(=O)C)cc1N |
| CHEMBL2002240 | N#Cc1c(N)cc(NC(=O)C)nc1-c(c2)ccc(c23)cccc3 |
| CHEMBL1966279 | NCCCNS(=O)(=O)c1cc(ccc1)C(=O)Nc(cc2)ccc2-c3n[nH]c(=O)c(c34)cccc4 |
| CHEMBL1998435 | OCCNc(c1)ncc(c12)ccc(c2)-c3ccncc3 |
| CHEMBL1989957 | OCCNc(c1)ncc(c12)ccc(c2)-c3ccccc3 |
| CHEMBL2004544 | O=S(=O)(O)c1ccc(c(c12)c(O)ccc2)NCc(c3)ccc(c34)OCO4 |
| CHEMBL1970074 | FC(F)(F)c1cc(ccc1)NC(=O)Nc(cc2)ccc2-c3cccc(c34)[nH]nc4C |
| CHEMBL1979318 | c1cccc(I)c1C2C(C#N)=C(N)Oc(c23)[nH]nc3C |
| CHEMBL1998414 | c1cc(Cl)ccc1CCNC(=O)c(c2)sc(c23)[nH]nc3C |
| CHEMBL1461728 | c1ccc(F)cc1C(=O)Nc(c2)ccc(c23)[nH]nc3 |
| CHEMBL2003638 | Fc1cccc(c1)C(=O)Nc(n[nH]2)c(c23)cc(cc3)-c4cn(nn4)Cc5ccccc5 |
| CHEMBL1992555 | c1cccc(c12)[nH]c(n2)Sc3oc(cc3)\C=N\NC(=O)c4cc([N+]([O-])=O)ccc4 |
| CHEMBL2000894 | c1cc(O)cc(C)c1-n2cc(C(=O)NN)c(=O)c(c23)ccc(c3)-c4ccncc4 |
| CHEMBL1983195 | c1ccoc1-c(n2)c(-c3occc3)nc(c24)ccc(c4)NC(=O)N(CC)CC |
| CHEMBL1986328 | c1ccoc1-c(n2)c(-c3occc3)nc(c24)ccc(c4)NC(=O)N(C)C |
| CHEMBL2006156 | c1ccoc1-c(n2)c(-c3occc3)nc(c24)ccc(c4)NC(=O)N(C(C)C)C(C)C |
| CHEMBL1997839 | c1cc(O)cc(C)c1-n2cc(C(=O)OCC)c(=O)c(c23)ccc(c3)-c4ccccc4 |
| CHEMBL1965631 | CN(C)CC(=O)Nc(n[nH]1)c(c12)cc(cc2)-c3cn(nn3)Cc4ccccc4 |
| CHEMBL1999931 | c1ccccc1C(=O)Nc(n[nH]2)c(c23)cc(cc3)-c4cn(nn4)Cc5ccccc5 |
| CHEMBL1990415 | c1cnccc1C(=O)Nc2n[nH]c(c23)nc4c(c3)cccc4 |
| CHEMBL1997025 | C1CCNCC1c(cc2)cc(F)c2-c(n3)[nH]c(c34)cccc4C(=O)N |
| CHEMBL1984711 | C1CCCCC1c(c2)noc2Nc(ccn3)nc3Nc(cc4OC)cc(OC)c4OC |
| CHEMBL1419458 | Nc1nonc1-n2nnc(c2-c3ccccc3)C(=O)N/N=C/c4ccncc4 |
| CHEMBL2004692 | c1ccsc1\C=N\NC(=O)c(c2-c3ccccc3)nnn2-c4nonc4N |
| CHEMBL2005631 | Nc1ncc(C(=O)NC)c(c12)scc2-c3ccc(cc3)NC(=O)Nc4c(F)ccc(c4)C |
| CHEMBL1964937 | c1ccccc1C(CO)NC(=O)N2CC=C(CC2)c3c[nH]c(c34)nccc4 |
| CHEMBL1984162 | Cc1cc(C)cc(c12)nc(o2)Nc(cc3)ccc3-c4nn(c(c45)ncnc5N)C6CCC(CC6)OCCOC |
| CHEMBL1983449 | c1cc(C)c(C)cc1CNc(c(=O)c2=O)c2Nc3ccncc3 |
| CHEMBL2003768 | c1cnccc1Nc2c(c(=O)c2=O)NCc3c(OC)cc(OC)cc3 |
| CHEMBL1966035 | c1cnccc1Nc(c(=O)c2=O)c2NC(C)c3ccccc3 |
| CHEMBL1982465 | C1COCCN1CC#Cc(cnc2N)c(c23)scc3-c4ccc(cc4)NC(=O)Nc5cc(C)ccc5 |
| CHEMBL1995736 | CCOC(=O)c1cccc(c12)[nH]c(n2)-c3ccc(cc3)N4CC(N)CC4 |
| CHEMBL1992242 | c1nsc(c12)cccc2-c3ccc(cc3)NC(=O)Nc4cc(C)cc(c4)C |
| CHEMBL2002165 | OC(C)Cn1ncc(c1)-c(cnc2N)c(c23)scc3-c4ccc(cc4)NC(=O)Nc5cc(F)ccc5 |
| CHEMBL1997822 | c1ccc(S(=O)(=O)C)cc1-c(cnc2N)c(c23)scc3-c(c4)ccc(c45)[nH]c(c5)C |
| CHEMBL1982383 | NCCc(ncc1)c(n12)nc(-c3ccc(F)cc3)c2-c4nc(ncc4)NCC(C)(C)CO |
| CHEMBL2006481 | c1n[nH]c(c12)ccc(c2)-c3onc(c3)C(=O)N4CCCCC4 |
| CHEMBL1970369 | c1n[nH]c(c12)ccc(c2)-c3onc(c3)C(=O)N4CC(C(=O)N)CCC4 |
| CHEMBL1082440 | c1ncc(C)c(c12)c(ccc2)S(=O)(=O)N3CCCNCC3C |
| CHEMBL1969879 | OCC(=O)N1CC(C(C)CC1)N(C)c2ncnc(c23)[nH]cc3 |
| CHEMBL392642 | CSc1ccccc1CNc2ncc([N+](=O)[O-])c(n2)NCC3CCC(CC3)CN |
| CHEMBL2006580 | Clc1csc(c12)ncnc2Nc3ccc(O)cc3 |
| CHEMBL1983309 | Clc(c1)ccc(c12)nc(s2)NC(=O)c3c(OC)cc(OC)cc3 |
| CHEMBL1966628 | c1ccnc(c12)[nH]cc2-c3nc(ncc3)NC4CCC(N)CC4 |
| CHEMBL2002446 | C1CC1COCC#Cc(sc2)cc2-c3n[nH]c(c34)c5c(C4)cc(cc5)CN6CCN(C)CC6 |
| CHEMBL1984039 | Cn1nc(C)c(c1C)-c2cccc(c23)c(c([nH]3)C(=O)O)CCCOc4cccc(c45)cccc5 |
| CHEMBL1968926 | c1ccccc1CSc([nH]n2)nc2-c3ccncc3 |
| CHEMBL2004615 | O=C(N)c(c1N)c(SC)nn1-c2ncccc2 |
| CHEMBL1971430 | c1ccc(C)cc1-c(c(=O)n2C)c(-c3ccncc3)nc2NCC(N)Cc4ccccc4 |
| CHEMBL2006010 | c1cc(Cl)c(Cl)c(c12)nccc2NC(=O)Nc(nc3C(F)(F)F)ccc3 |
| Inactives: |  |
| CHEMBL351859 | CC1(C)CCC(C)(C)c2cc(ccc12)c3c(C4=CC(=O)NC4=O)c5ccccc5n3CCCSC(=N)N |
| CHEMBL3818367 | CC(C)C(O)(c1ccc(C#N)c(Cl)c1Cl)C(F)(F)F |
| CHEMBL228442 | COc1cc(ccc1OCCn2c3ccccc3c4ccccc24)C5NC(=O)NC(=C5C(=O)O)C |
| CHEMBL17495 | COc1cc(ccc1OCCCN2CCC(CC2)C(O)(c3cccc(F)c3)c4cccc(F)c4)C(=O)C |
| CHEMBL3343442 | CC(=O)OCC1=CC(=O)N(O)C(=C1)Cc2ccccc2 |
| CHEMBL446107 | CN1SC(=NC1=O)NCC(=O)O |
| CHEMBL445395 | OCC1O[C@@H](O\N=C\c2occc2)C(O)[C@@H](O)C1O |
| CHEMBL2371745 | CCCCC(C)C(O)CC(=O)N[C@@H](Cc1ccccc1)C(=O)N[C@@H](C)C(=O)N[C@@H](CC(C)C)C(=O)O |
| CHEMBL3673774 | NC(=O)n1cc(NC(=O)N2C[C@H](F)C[C@H]2C(=O)Nc3cc(cc(Br)c3F)C(=O)O)c4ccccc14 |
| CHEMBL2017124 | CC(=O)Nc1ccc(NC(=O)C(=O)O)c(c1)C(=O)c2ccccc2 |
| CHEMBL3941394 | Cn1nc(cc1CN2CCN(CC2)C(=O)OC(C(F)(F)F)C(F)(F)F)c3ccccc3 |
| CHEMBL3972436 | COc1ccc(CC(=O)O)cc1c2ccc(F)c3CCN(Cc23)C(=O)CC4CCOc5ccccc45 |
| CHEMBL45717 | Cc1cccc(NCC2=Nc3ccc(F)cc3C(=O)N2c4ccccc4Cl)c1 |
| CHEMBL1270285 | COc1cc(cc(OC)c1OC)C(=O)c2ccc(cc2n3cncn3)c4csc(NC(=O)[C@@H]5CCCN5)n4 |
| CHEMBL112699 | CCOc1ccc(CNc2nnc(Cl)c3ccc(cc23)C#N)cc1Cl |
| CHEMBL3408645 | CC(=O)Nc1ccc(cc1)C(=O)C |
| CHEMBL3682160 | O=C(N1CC(C1)c2nccnc2c3ccccc3)c4nc5ccccc5[nH]4 |
| CHEMBL1940123 | O=C(CCNS(=O)(=O)c1cccc2scnc12)N3CCN(CC3)c4ccncc4 |
| CHEMBL3682886 | COc1ccc(nc1c2ccc(F)c(F)c2)C(=O)N[C@@H](CC(=O)O)c3ccc(C)cc3 |
| CHEMBL2088432 | COc1ccc(CNc2nc(NC[C@H](O)CO)nc3c(NCc4ccc(OC)cc4)nc(NC[C@H](O)CO)nc23)cc1 |
| CHEMBL1829526 | CC(=O)O[C@@H]1C[C@](C)(O)[C@@]23C[C@@H](C[C@H](OC(=O)c4cocc4)[C@]2(C)[C@H]1OC(=O)c5cocc5)C(C)(C)O3 |
| CHEMBL3890693 | CC(C)(C)OC(=O)N1C[C@H](C[C@H]1C(=O)N[C@@H](Cc2ccc(OCc3ccc(cc3)[N+](=O)[O-])cc2)C(=O)NS(=O)(=O)c4ccc(Cl)c(c4)[N+](=O)[O-])Oc5ccc(cc5)c6ccccc6 |
| CHEMBL144638 | CC1(C)Oc2ccc(cc2[C@H]([C@@H]1O)N3C=C(Br)C=C(Br)C3=O)C#N |
| CHEMBL2358292 | O[C@H]1COC[C@@H]2O[C@@H](CC(=O)Nc3ccccc3)CC[C@H]2N(C1)S(=O)(=O)c4cccc(F)c4 |
| CHEMBL15390 | CCCOc1ccc(OC)cc1c2ncc(c(O)n2)c3nn[nH]n3 |
| CHEMBL245542 | Clc1ccc(cc1)S(=O)(=O)N2CC(=O)NC\C(=C/c3ccccc3)\C2=O |
| CHEMBL3229487 | OC[C@H]1O[C@@H]2[C@H](OC3=NC(=N)C(=CN23)F)[C@@H]1OP(=O)(O)O |
| CHEMBL1629918 | COc1cc(C=O)ccc1OC(=O)c2cn(nc2c3ccc(F)cc3)c4ccccc4 |
| CHEMBL3703929 | CC(=C)c1ccc(Cc2cc([C@@H]3O[C@H](CO)[C@@H](O)[C@H](O)[C@H]3O)c4CCOc4c2Cl)cc1 |
| CHEMBL3184995 | CC(=O)C(Cc1ccccc1)NC(=O)COC(=O)c2cc(ccc2F)S(=O)(=O)N3CCOCC3 |
| CHEMBL3907126 | CC[C@@H](N[C@@H](C)CC(=O)N)c1ccc(Cl)c(C(=O)c2ccns2)c1F |
| CHEMBL3229757 | NC(=O)c1cc(C(O)CC2CCCCN2)c3cccc(c3n1)C(F)(F)F |
| CHEMBL157142 | CC1=CN(C2CC(O)C(NC(=O)CC3CC(OC3CO)n4cnc5c(O)nc(N)nc45)O2)C(=O)NC1=O |
| CHEMBL3665338 | CC(C)N1CCCc2c(C1)c3ccc(cc3n2C)N4C=CC(=CC4=O)OCc5ccccc5 |
| CHEMBL179525 | C[C@](O)(CI)C(=O)Nc1ccc(c(c1)C(F)(F)F)[N+](=O)[O-] |
| CHEMBL90628 | COc1ccc(CNc2nc(NC(O)CN)nc3c2ncn3C(C)C)cc1 |
| CHEMBL441603 | NC(=N)c1ccc(cc1)N2CCN(Cc3cccc(CC(=O)O)c3)CC2 |
| CHEMBL3248101 | CC(C)NCC(O)COc1ccc(cc1)S(=O)(=O)c2ccc(Cl)cc2 |
| CHEMBL170363 | CSc1sc(cc1c2csc(Nc3cccc(OCC(=O)N)c3)n2)C(=N)N |
| CHEMBL1163675 | CCOC(=O)\C=C\c1cn(nc1c2ccccc2)c3ccc(O)cc3 |
| CHEMBL3673627 | C[C@@H]1CNC(=O)c2cc3ccc(nc3n12)C(=O)Nc4cnc5ccccc5c4 |
| CHEMBL1939039 | CCC1=CC(=O)OC2=C1C(=O)NC(=N2)OCC#CC3CC3 |
| CHEMBL224610 | N[C@@H](Cc1ccccc1)C(=O)N2CCC[C@H]2C(=O)N |
| CHEMBL3895719 | Clc1cncc(Cl)c1NC(=O)c2ccccc2Sc3ccc4c(\C=C\c5ccccn5)n[nH]c4c3 |
| CHEMBL3236156 | Fc1cccc(CCN=[N+]=[N-])c1 |
| CHEMBL108206 | CNc1ccc(Cc2ccc(CCCCCCC(=O)O)cc2)cc1 |
| CHEMBL3187732 | COC1=CC(=O)N2CCN(Cc3ccc(F)cc3)CCC2=C1C(=O)N(C)Cc4nonc4C |
| CHEMBL3684798 | CCC(C[C@H]1COC(=N1)N)c2ccc(cc2)C(F)(F)F |
| CHEMBL2133701 | C[C@@H](CO)N1C[C@H](C)[C@@H](CN(C)Cc2ccc(cc2)C(=O)Nc3ccccc3N)Oc4ccc(NC(=O)Nc5cccc6ccccc56)cc4C1=O |
| CHEMBL3085211 | CCCCC[C@H](O)\C=C\C=C/C\C=C\CCCCC(=O)O |
| CHEMBL3315320 | CC(C)C[C@H](NC(=O)CCc1ccccc1)C(=O)N[C@@H](Cc2ccccc2)C(=O)N[C@@H](CCNC(=N)N)C(=O)N3CCC[C@H]3C(=O)N[C@@H](CCCNC(=N)N)C(=O)N[C@@H](CC(=O)N)C(=O)N |
| CHEMBL488983 | O=C(CCNC(=O)c1nc2ccccc2n1Cc3ccccc3)N4CCN(CC4)c5ccncc5 |
| CHEMBL141468 | CN(C)Cc1cccc(c1)C(=O)c2oc3cc(cc(O)c3c2C)c4ccccc4 |
| CHEMBL3715605 | OC(=O)C(F)(F)F.Fc1ccc(cc1C(=O)C2CCN(CC2)c3nc4cnccc4nc3NC5CC5)C#N |
| CHEMBL464537 | COc1cc(cc(OC)c1OC)C2=CC3=C(C[C@@]4(O)[C@@](C)(CC[C@@]5(O)[C@](C)(CO)C=CC(=O)[C@]45C)O3)C(=O)O2 |
| CHEMBL9966 | Clc1ccccc1CN2CCC(CC2)C3(CCC(=O)NC3=O)c4ccccc4 |
| CHEMBL2323256 | CC(OCc1ccccc1)C(NC(=O)CC23CC4CC(CC(C4)C2)C3)C(=O)N5CCN(Cc6ccccc6)CC5 |
| CHEMBL73613 | OC(=O)[C@@H]1CCC[C@@H](N1N=O)C(=O)O |
| CHEMBL136236 | CCNCCOc1cccc2ccccc12 |
| CHEMBL3187624 | COc1ccc(cc1)N2CCN(CC2)C(=O)C3CCN(CC3)C(=O)c4ccccc4Cl |
| CHEMBL1593789 | OCCCCC\C=C/C[C@@H](O)\C=C\C=C\C=C/[C@@H](O)CCCC(=O)O |
| CHEMBL2136485 | C[C@H](CO)N1C[C@H](C)[C@H](CN(C)S(=O)(=O)c2ccc(C)cc2)OCc3cnnn3CCCC1=O |
| CHEMBL2218653 | CC(C)(C)c1ccccc1N2CCN(Cc3ccc(F)cc3Cl)C(=O)C2=O |
| CHEMBL4094925 | NS(=O)(=O)Oc1ccc2C=C(C(=O)Oc2c1)c3ccc(NC(=O)Cc4ccccc4)cc3 |
| CHEMBL203446 | CC1C2CCC1C=C(C2)C=C3c4ccccc4CCc5ccccc35 |
| CHEMBL4087890 | COc1cccc(COc2nc(ccc2CNC(=O)C(C)c3ccc(NS(=O)(=O)C)c(F)c3)C(C)(C)C)c1 |
| CHEMBL2361412 | COC1=CC(=O)N2CCN(Cc3ccccc3C(F)(F)F)CCC2=C1C(=O)NC(C)c4occc4 |
| CHEMBL254922 | CCCCN(CCCC)CCCNc1nc(NCCCN(CCCC)CCCC)nc(NC23CC4CC(CC(C4)C2)C3)n1 |
| CHEMBL1923302 | CCN1C(=O)c2nc(c3ccc(cc3)c4ccccc4)n(Cc5ccc(F)cc5)c2N6C[C@H](N=C16)C(C)C |
| CHEMBL472591 | COc1cccc2C=C(C(=O)N)\C(=N\c3ccc(cc3)c4ccccc4)\Oc12 |
| CHEMBL3903284 | Brc1cc(ccc1Nc2cc(NC3CC3)n4ncc(\C=C\5/NC(=O)NC5=O)c4n2)C#N |
| CHEMBL384134 | CCc1ccc2c(C(=O)O)c(O)c(Cc3ccc(Cl)cc3)nc2c1 |
| CHEMBL1289398 | CC[C@H]1O[C@H]([C@H](O)[C@@H](O)[C@@H]1O)c2ccc(Cl)c(Cc3cnc(nc3)c4nncs4)c2 |
| CHEMBL3716480 | OC(=O)C(Cc1ccc(OCc2ccc(cc2)C#N)cc1)NC(=O)c3cn4ccc(cc4n3)c5ccc(Cl)cc5 |
| CHEMBL3908477 | Cn1ncc(C(=O)N2CCC2)c1C(=O)Nc3ccc4nc(nn4c3)N5CCOCC5 |
| CHEMBL3718381 | CC(C)N(C)c1nc2cc(ccc2nc1c3ccccc3F)C(=O)O |
| CHEMBL468089 | CCc1ccc(cc1)S(=O)(=O)N[C@H]2[C@H](O)CCc3ccc(NC(=O)CNc4cccc(C)c4)cc23 |
| CHEMBL30101 | CCNC(C)C(O)COc1ccc(CC(=O)N)cc1 |
| CHEMBL3967656 | CN(Cc1cc(NC(=O)c2sc3c(ccc(C(=O)C)c3c2C)C(=O)C)nc4ccccc14)C(=O)C |
| CHEMBL3911894 | NC(=O)N1CCCc2cc(cnc12)c3cncc(c3)C(=O)N4CCOCC4 |
| CHEMBL3612916 | FC(F)(F)Oc1ccc(cc1)S(=O)(=O)CCCN2c3ccccc3CCc4ccc(Cl)cc24 |
| CHEMBL3695309 | C[C@H](NC(=O)C)c1ccc(O[C@@H]2CCN(C2)c3cccc(n3)N4CCOCC4C)cc1 |
| CHEMBL3580937 | C\C=C/1\C[N@@+]2(C)CC[C@@H]1[C@H](C(=O)[O-])c3[nH]c4ccccc4c3CC2 |
| CHEMBL416700 | COc1ccc(CCCN2CCC(CCC(c3ccccc3)c4ccccc4)CC2)cc1 |
| CHEMBL525187 | COC(=O)c1cc(NC(=O)C(C)Br)cc(c1)C(=O)NC(=O)N |
| CHEMBL307311 | COc1cccc(c1)n2ncc3c(N\N=C\c4ccccc4F)ncnc23 |
| CHEMBL1909778 | Cl.NC(=N)NC(=O)c1nc(Cl)cnc1N |
| CHEMBL1802491 | C(CN1CCOCC1)Nc2nccc(n2)c3c([nH]c4ccccc34)c5ccccc5 |
| CHEMBL366178 | CCC(C)[C@H](NC(=O)CCCCCCCCCCCCCCC(=O)N[C@@H](CC(=O)N)C(=O)NC(Cc1ccc2ccccc2c1)C(=O)O)C(=O)N[C@H](Cc3ccccc3)C(=O)N |
| CHEMBL2088642 | FC(F)(F)c1cccc(c1)C(=O)NC2CCCCC2 |
| CHEMBL185096 | CCC(CC)Oc1cccc2ccc(N)nc12 |
| CHEMBL251419 | CCCCCCCN(C1Cc2ccc(SC(C)(C)C(=O)O)cc2C1)C(=O)Nc3ccccc3SC(F)(F)F |
| CHEMBL1831095 | CC1=C(C(c2ccc(cc2)[N+](=O)[O-])c3c(O)ccc4ccccc34)C(=O)N(N1)c5ccccc5 |
| CHEMBL1554514 | Clc1ccccc1CNc2nc(nn2C(=O)c3ccccc3)c4occc4 |
| CHEMBL513087 | C[C@H](CS)C(=O)N[C@@H](CCSCc1ccc(cc1)N(C)C)C(=O)O |
| CHEMBL2164441 | CS(=O)(=O)c1ccc(cc1Cl)C(CC2CCCC2)C(=O)Nc3nccs3 |
| CHEMBL125352 | CC(=O)Nc1ncc(SCc2csc(NC(=O)C)n2)s1 |
| CHEMBL2440843 | OC(COc1ccc2c(c1)[nH]c3ccccc23)CN4CCOc5ccccc45 |
| CHEMBL425685 | Cc1onc(CN2CCCC(CNc3nc(N)n4nc(nc4n3)c5occc5)C2)c1 |
| CHEMBL3350372 | CC(C)COC(=O)[C@H](NC(=O)[C@@H](N)CC(=O)O)[C@H](C)O |
| CHEMBL4066746 | CCOC(=O)c1csc(n1)c2csc(n2)c3csc(n3)[C@@H](O)Cc4ccccc4 |
| CHEMBL246917 | Nc1ccc(cc1)S(=O)(=O)N2CCOc3c(cc(F)cc23)N4CCNCC4 |
| CHEMBL4086916 | CN(C)[C@H]1CN(Cc2c(F)cccc2Br)C[C@@H]1C3CC3 |
| CHEMBL1223308 | Clc1ccccc1C(N2CCCN(CC2)C3CCC3)c4nnnn4Cc5ccccc5 |
| CHEMBL3359567 | Cl.CN1CCCN(CC1)c2ccc(cc2)C(=O)Nc3ccccc3C(=O)Nc4nc5ccccc5[nH]4 |
| CHEMBL3662574 | Fc1cc(ccc1C[C@H](NC(=O)[C@H]2N[C@@H]3CC[C@H]2C3)C#N)c4cnc(s4)N5CCOCC5 |
| CHEMBL3682609 | Cc1c(nc(C2CC2)c3ccccc13)N(Cc4ccc(F)c(c4)C(F)(F)F)S(=O)(=O)c5ccc(cc5)C(=O)O |
| CHEMBL353270 | CN(N=O)c1ccc(cc1)N=O |
| CHEMBL2205459 | COc1cc(Nc2ncc(C#N)c(NC3=C(N[C@H](C)C(C)(C)C)C(=O)C3=O)n2)cc(OC)c1OC |
| CHEMBL3967868 | C[C@H](NC(=O)[C@H]1CCC2CC2N1Cc3ccc(cc3)C(F)(F)F)c4ccc(cc4)C(=O)O |
| CHEMBL3965292 | Cc1onc(C)c1Cn2cc(cn2)N3C(=O)CN(Cc4ccccc4F)C3=O |
| CHEMBL3277217 | CCCCCCCCCCN1CCCC(C1)C(=O)N |
| CHEMBL3785439 | COc1ccc(cc1)C(=O)NC[C@H]2O[C@H]([C@H](O)[C@@H](O)[C@@H]2O)n3cc(Cc4ccc(cc4)C5CC5)c6c(Cl)cccc36 |
| CHEMBL3318420 | OCc1ccccc1SC2=C(O)CC(CC2=O)c3ccccc3 |
| CHEMBL142570 | CC(C)[C@H](NC(=O)OCc1ccccc1)C(=O)N[C@@H](Cc2ccccc2)[C@@H](O)[C@@H](NCc3ccccc3)C(=O)N[C@@H](C(C)C)C(=O)NCc4ccncc4 |
| CHEMBL1089066 | C[C@]12CC[C@H]3[C@@H](CCC4=CC(=O)CC[C@]34C)[C@@H]1CC[C@@H]2C(=O)COS(=O)(=O)c5ccccc5 |
| CHEMBL134111 | C[C@H](C(NC(=O)C1CCCN(C1)S(=O)(=O)c2cccs2)C(=O)N[C@@H](CCCCN)C(=O)OC(C)(C)C)c3c[nH]c4ccccc34 |
| CHEMBL210460 | CC(C)C(C\C=C/1\CC(CO)(COC(=O)c2ccccc2O)OC1=O)C(C)C |
| CHEMBL471263 | CCCCCCCCOc1ccc(cc1F)c2c[nH]c(n2)[C@@](C)(N)CO |
| CHEMBL3664034 | Nc1cc(F)ccc1NC(=O)c2cnc3cc(ccc3n2)N4CCNCC4 |
| CHEMBL3741212 | CCn1c(CNc2ccc(cc2)C(=N)N)nc3cc(ccc13)C(=O)N(CCC(=O)O)c4ccc(F)cc4 |
| CHEMBL1903432 | CC[C@]1(N[C@H](CN(C)C(=O)Nc2ccc(Cl)cc2)[C@H]3[C@@H]1C(=O)N(C)C3=O)C(=O)OC.OC(=O)C(F)(F)F |
| CHEMBL3658604 | O[C@H]1C[C@@H]2C[C@H]1[C@H](N2)C(=O)N[C@@H](Cc3ccc(cc3)c4ccc(C#N)c(F)c4)C#N |
| CHEMBL4065683 | CC(C)C[C@@H](CC(=O)O)NC(=O)CC[C@@H](C)[C@H]1CC[C@H]2[C@@H]3CC[C@@H]4C[C@H](O)CC[C@]4(C)[C@H]3CC[C@]12C |
| CHEMBL4110325 | CCCC(=O)N1CCC(C1)C(=O)N2CC[C@@]3(C)c4cccc(O)c4C[C@@H]2C3(C)C |
| CHEMBL492181 | FC(F)(F)c1ccccc1C(=O)C2=Cc3c(OC2=O)ccc4ccccc34 |
| CHEMBL3938089 | O=C(Nc1ccc(cc1)C(=O)NCc2cccnc2)N3Cc4ccncc4C3 |
| CHEMBL1885040 | Cc1cnc(cn1)C(=O)N2CCN(CC2)S(=O)(=O)c3ccc(F)c(F)c3F |
| CHEMBL388228 | Cc1cc(ccc1NC(=O)C(F)(F)F)c2cn3cc(Br)ccc3n2 |
| CHEMBL2414790 | O=C(Nc1ccc(cc1)c2cn3c(CN4CCNCC4)csc3n2)c5cnc6ccccc6n5 |
| CHEMBL1088869 | CC(C)C(=O)O[C@@H](C=C)c1ccc(OC(=O)C)cc1 |
| CHEMBL1858973 | CSc1ccccc1Oc2ncccc2\C(=N\O)\NCc3cccs3 |
| CHEMBL280739 | CC[C@@]1(OC[C@@H](O1)[C@@H]2CCCCN2)c3cccc(Cl)c3 |
| CHEMBL252047 | Nc1ccccc1NC(=O)c2cc3ccc(cc3s2)C(NCCc4ccncc4)C(=O)NCc5ccccc5 |
| CHEMBL2361177 | C[C@H](CO)N1C[C@H](C)[C@H](CN(C)Cc2ccc(cc2)C(=O)Nc3ccccc3N)Oc4c(NC(=O)Nc5ccccc5)cccc4C1=O |
| CHEMBL3941239 | COc1ncccc1c2cncc(c2)C(=O)Nc3ccc(OC(F)(F)F)cc3 |
| CHEMBL3979581 | OC(=O)c1[nH]c2cc(Cl)ccc2c1C(N(Cc3ccccc3)C=O)C(=O)NC4CCCCC4 |
| CHEMBL4112396 | OC[C@H]1C[C@@H](NC2=C(C(=O)NC(=N2)N3CCC(CC3)c4cncnc4)c5nc6ccccc6s5)[C@H](O)[C@@H]1O |
| CHEMBL3941648 | O=S(=O)(Nc1cccc2cccnc12)c3cccnc3 |
| CHEMBL3733087 | COc1cccc(c1)C#Cc2ccc3nnc(c4cccc(OC(F)(F)F)c4)n3c2 |
| CHEMBL1288643 | Oc1ccc(cc1O)C2COc3c(O)c(O)ccc3C2 |
| CHEMBL2296465 | COc1ccc2nc(\N=C\c3ccc(cc3)[N+](=O)[O-])sc2c1 |
| CHEMBL1812041 | C\C(=C\1/C(=O)[C@@H]2[C@@H]3[C@H](Cc4cccc5[nH]cc3c45)C(C)(C)N2C1=O)\O |
| CHEMBL3654617 | COc1cccc(F)c1CN2C[C@@H](C)C[C@H](C2)NC(=O)c3ccc4[nH]nc(c5ccn6ncnc6c5)c4c3 |
| CHEMBL3982231 | Nc1ccc(CNC(=O)c2ccc(NC(=O)N3Cc4cccnc4C3)cc2)cc1 |
| CHEMBL2334676 | CC(C)C[C@H](N)C(=O)N1[C@@H](C[C@@H]2C[C@H]12)C#N |
| CHEMBL3915010 | Cn1cc(cn1)c2cnc3nnn(C[C@@H]4CN(CCO4)c5ncc(cn5)c6cnn(c6)C7CCNCC7)c3n2 |
| CHEMBL330663 | Cn1cnc2nncc2c1S |
| CHEMBL1911459 | OC(CNCc1ccc(F)cc1F)Cn2c3CCCCc3c4ccccc24 |
| CHEMBL431574 | Clc1ccc(CN2CCCC2)cn1 |
